# Comparative analyses of co-evolving host-parasite associations reveal unique gene expression patterns underlying slavemaker raiding and host defensive phenotypes

**DOI:** 10.1038/s41598-018-20262-y

**Published:** 2018-01-31

**Authors:** Austin Alleman, Barbara Feldmeyer, Susanne Foitzik

**Affiliations:** 1 Institute of Organismic and Molecular Evolution, Johannes Gutenberg University Mainz, Johannes von Müller Weg 6, Mainz, 55128 Germany; 2 Senckenberg Biodiversity and Climate Research Centre, Senckenberg Gesellschaft für Naturforschung, Senckenberganlage 25, D-60325 Frankfurt am Main, Germany

## Abstract

The transition to parasitism is a drastic shift in lifestyle, involving rapid changes in gene structure, function, and expression. After the establishment of antagonistic relationships, parasites and hosts co-evolve through reciprocal adaptations, often resulting in evolutionary arms-races. Repeated evolution of social parasitism and slavery among *Temnothorax* ants allows us to examine those gene expression patterns that characterize slavemaker raiding and reciprocal host defensive phenotypes. Previous behavioural studies have established that raiding strategies between *Temnothorax* slavemakers diverge, while host defense portfolios shift similarly under parasite pressure. We are the first to confirm this at the molecular level, revealing that slavemaking species exhibit a wider variety of genes with species-specific patterns of expression within their raiding phenotypes, whereas expression similarity is commonly found during the non-raiding phenotype. Host species response to slavemaker aggression, however, is indicated by strong changes in the expression of a relatively few number genes. Additionally, the expression of individual genes such as *Acyl-CoA-Delta(11) desaturase* and *Trypsin-7* is strongly associated with the raiding phenotype of all three slavemaking species. Here, we provide novel insight into the gene expression patterns associated with raiding and nest defense behavior in *Temnothorax* ants, suggesting lineage-specific evolutionary patterns among both slavemakers and hosts.

## Introduction

Understanding the processes that shape the evolutionary trajectories of organisms is a long-standing goal of the biological sciences. Parallel and convergent evolutionary patterns are of particular interest, raising questions as to the predictability and repeatability of evolution. Understanding the molecular mechanisms leading to the repeated evolution of similar phenotypes allows for the elucidation of factors that shape biological diversity. Phenotypic convergence can arise through many molecular mechanisms, where similarities can occur at a number of different hierarchical levels (nucleotide, gene, pathway, etc.)^1^. As genetic constraints can strongly influence probability of convergent or parallel evolution, the occurrence similar phenotypes is more likely within closely-related lineages containing similar genetic and molecular repertoires. Co-evolution among parasites and their hosts offers a unique and ideal system in which to investigate how convergent and parallel evolution affect ecological diversification^2^. Relatedness between parasite and host can vary widely between taxa. While some parasites are only distantly related to their hosts, such as viruses and their human hosts, many parasites – such as avian brood parasite – share close phylogenetic ties^3^. There are few systems where closely-related parasites and hosts occur as frequently as in social insects. Characterized by variable phenotypes and complex social systems, insect societies are particularly susceptible to exploitation by closely-related taxa^4,5^. Often occurring as a mechanisms of circumventing the cost of parental care, social parasitism is widespread within bees^6–12^ and ants^13,14^, and not uncommon within wasps^15–17^.

Within the ant genus *Temnothorax*, social parasitism has evolved multiple times independently^18–20^. As such, many characteristics ideal for investigating the molecular basis of phenotypic traits associated with social parasitism may be found within this genus. (1) *Social parasites are often closely-related to their hosts, and as such share genetic ancestry*; and *Temnothorax* is no exception. One small North American clade is comprised of three slavemakers (*Temnothorax americanus*, *T. pilagens*, and *T. duloticus*) and their three closely related host species (*T. ambiguus*, *T. longispinosus*, and *T. curvispinosus*). The obligate slavemaking species of this taxon, *T. americanus* (genus name recently changed from *Protomognathus*^21^), *T. duloticus*, and *T. pilagens*, all display active raiding and slavemaking behaviour^22–24^.

(2) *Socially-parasitic species have lost many traits essential for a free-living lifestyle*. Slavemakers reside within their own mixed-species nests, but carry out destructive ‘*slave raids*’ against nearby host colonies in order to steal brood and thus strengthen their own workforce^4^. From these stolen host larvae and pupae, a new generation of slaves will develop, which will subsequently carry out all routine worker tasks – such as brood-care and foraging - in the slavemaker nest. In stark contrast, slavemaker workers have almost completely lost the ability to work, and instead are highly specialized for raiding^25^. Slavemaker workers and queens alike have developed mechanisms to subvert, disrupt, or otherwise bypass ordinary host recognition and communication systems^26–29^. In addition to slavemaker-specific morphological characteristics such as powerful mandibles^22,23^, potent stingers^23,24^, and enlarged petioles^25^, slavemakers often employ the Dufour’s gland and other glandular secretions to manipulate hosts^30,31^, mimic host profiles^32^, or obtain recognition cues directly from hosts in order to camouflage themselves^33^.

**Figure 1:**
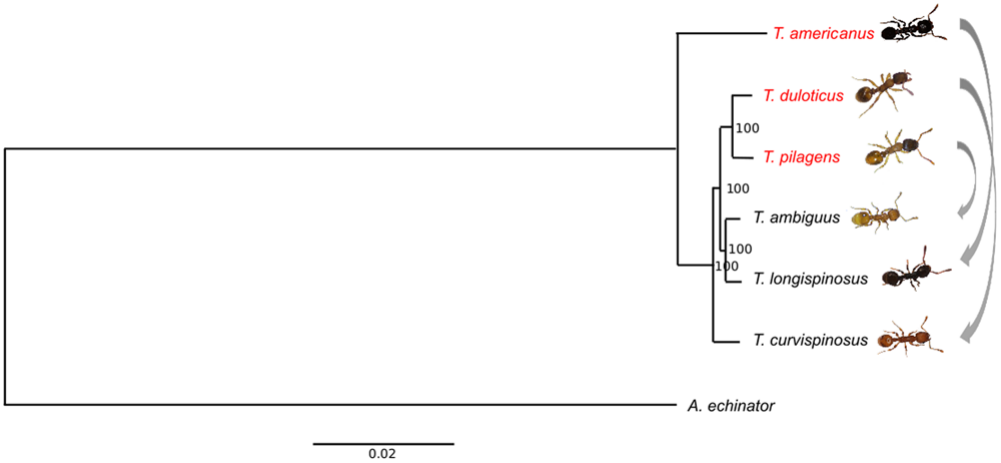
Maximum Likelihood phylogenetic relationship between the six *Temnothorax* species herein examined, with *Acromyrmex echinatior* as outgroup (used with permission^19^). Produced using RaxML based on 5199 orthologous gene clusters (ML boostrap values given at each node). Slavemaker *T. americanus* preferentially parasitizes *T. longispinosus*, *T. duloticus* preferentially parasitizes *T. curvispinosus*, and *T. pilagens* preferentially parasitizes *T. ambiguus*.

Indeed, this rapid diversification of species-specific mechanisms and strategies within social parasites is a direct indication of (3) *increased phenotypic diversity*, *a result of loosened phenotypic constraints associated with the transition away from a free-living lifestyle*^34^. The most phylogenetically distant *Temnothorax* slavemaker, *T. americanus* – which split from its non-parasitic ancestors between 22 and 12 million years ago^20^ – is also the most behaviourally and morphologically distinct (Fig. 1). This social parasite is able to exploit all three host species within this clade, *T. longispinosus*, *T. ambiguus*, and *T. curvispinosus;* though it clearly prefers *T. longispinosus*^35^. *T*. *americanus* utilizes glandular secretions to manipulate host defenders^23,30,31^, whereas raids conducted by *T. duloticus*, primarily against *T. curvispinosus*, tend to be more destructive since *T. duloticus* workers are much more prone to stinging host defenders to death^23,36–38^. *T. pilagens* preferentially raids *T. ambiguus* colonies, though will opportunistically target *T. longispinosus* as well^39^. Unique among *Temnothorax* slavemakers, raids by *T. pilagens* often remain peaceful, as hosts appear to be unable to recognize invading *T. pilagens* raiders^24^. In such cases, slavemakers are even able to lead adult host workers into slavery. In those raids where it is recognized as an enemy, *T. pilagens* responds violently to host attacks, stinging defending hosts and imparting high mortality upon the host colony^24^.

Presented with ever-evolving mechanisms of subversion and aggression by slavemaking species, hosts respond through defensive mechanisms that minimize the loss of individuals to raiding or aggression^40–42^, highlighting that (4) *parasite and host are engaged in an evolutionary arms race*. During slave raids, host colonies not only lose their brood, but workers and queens often die during defense of the colony. Accordingly, these raids can exert high fitness costs upon host colonies^36,43–45^. This results in continuous adaptation and counter-adaptation on both sides, with parasites having the advantage in some cases^46,47^, and hosts in others^46,48^.

Additionally, *Temnothorax* slavemakers and hosts alike display complex behaviours that are easily observed and recorded in a laboratory setting. This facilitates smooth integration of both molecular and phenotypic studies. These above factors, taken together, allow for effective elucidation of the molecular components underlying the behaviour and physiology of organisms with shared evolutionary history, as well as the examination of similarities and differences in phenotypes arising within the same environmental context.

The objectives for this project are informed by three primary assumptions about the molecular evolution of social parasitism, first outlined by Cini *et al*. (ref.^32^): (1) Firstly, *trait novelty or commonality will be reflected at the molecular level*. Within closely-related species, phenotypic diversification often operates through gene-regulatory shifts, rather than sequence alterations to protein-coding genes^49,50^. (2) *Novel molecular processes underlie lineage-specific phenotypes*. While groups of conserved genes associated with convergent social behaviours have been found in a number of eusocial insects^51–54^; more recent work has also revealed that eusocial lineages can harbor novel genes that are associated with eusocial behaviours^55–58^. (3) Lastly, that *conserved regulatory processes underlie the response to a shared environment*. In contrast to raiding behaviour, *Temnothorax* slavemakers display much more behavioural similarity when out of raiding season, universally possessing a reduced capacity for normal nest-work and a less active lifestyle. Given this, we might expect that gene regulation in this behavioural phenotype may indeed be more similar between species, certainly more so than expression patterns during the raiding phenotype. Here, we utilized an RNA-Seq approach following behavioural experiments in order to identify regulatory patterns involved in slavemaker raiding behaviour and host defensive behaviour by focusing on the three slavemakers (*T. americanus*, *T. pilagens*, and *T. duloticus*) and their three preferred host species (*T. ambiguus*, *T. longispinosus*, and *T*. *curvispinosus*). Workers of these species were collected during two different behavioural phenotypes: a raiding and a non-raiding phenotype. For hosts, the raiding phenotype is typified by active nest defense of hosts against a slavemaker raid, and the non-raiding phenotype characterized by a normal, non-antagonistic nest-life. For slavemakers, the raiding phenotype is typified by active slave raiding behaviour aimed at subverting host defenses and stealing host brood, and the non-raiding phenotype characterized by slavemakers within their own nests outside of raiding season. Workers of host species were collected (1) before any contact with slavemakers, and (2) during active nest defense from raiding slavemakers; and slavemakers workers collected (1) out of raiding season, and (2) during a slavemaking raid. The use of separate behavioural phenotypes allows for the disentanglement of intra-species (between behavioural phenotypes of a single species) and inter-species (within behavioural phenotypes across species) signatures of differential gene expression. Thus, by comparing three slavemaker and three host species, we were able to elucidate whether or not raiding or defensive strategies evolved along independent, species-specific trajectories, or whether these behaviours evolved in parallel within this genus.

## Results

### Transcriptome Sequencing and Assembly

In total, we obtained 1.16E + 09 raw reads across all six species, with an average of 24-million raw reads per replicate (Supplementary Table S1). Finalized transcriptomes vary from 43,664 contigs (*T. ambiguus*) to 79,227 contigs (*T. curvispinosus*), with N50 values ranging from 2,973 to 3,606 (Supplementary Table S2). The *T. ambiguus* transcriptome is the smallest, whereas the *T. curvispinosus* transcriptome is the largest. Total number of contig BLAST annotations varied between 16,433 and 31,636, with single gene annotations ranging from 10,206 in *T. pilagens* to 18,396 in *T. curvispinosus* (Supplementary Tables S1 and S2).

### Gene Expression and Weighted Gene Co-Expression Network Analyses

DEGs were determined to be up-regulated in either one of two phenotypes: a *raiding* and a *non-raiding* phenotype. A total of 3,381 genes were found to be differently-expressed between these two phenotypes of the slavemakers (*T. americanus*: 975, *T. duloticus*: 890, and *T. pilagens*: 1616; Fig. 2) and 697 genes differentially-expressed within the hosts’ two phenotypes (*T. longispinosus*: 209, *T. curvispinosus*: 108, and *T. ambiguus*: 380; Fig. 2; complete lists contained in Supplementary Tables S3 and S4). Examination of each slavemaker-host pair revealed that the ratio of DEGs from all expressed genes was higher in slavemakers than their preferred host species (*T. amer - T. longi:* χ² = 531.1; p < 0.0001; *T. dul – T. curvi:* χ² = 1133.6; p < 0.0001; *T. pila – T ambi*: χ² = 1916.8; p < 0.00001). Slavemaking species also displayed fewer genes up-regulated during raids when compared to the non-raiding phenotype (*T. amer:* χ² = 20.9; p < 0.0001; *T. dul:* χ² = 49.2; p < 0.00001; *T. pila*. χ² = 81.9; p < 0.00001). In contrast, however, we found no difference in the ratio of DEGs between phenotypes within two hosts, and, in the case of *T. longispinosus*, an *increased* number of genes up-regulated during nest defense (*T. longi:* χ² = 7.4 p < 0.01; *T. ambi*. χ² = 0.3; p = 0.56; *T. curvi*. χ² = 0.3; p = 0.58). Additionally, hosts possess a greater proportion of genes up-regulated during their defense when compared to the number of genes up-regulated during the slavemakers raiding phenotype (χ² = 86.6, p < 0.0001). Lifestyle-specific dendrograms produced during weighted gene co-expression network analysis (WGCNA) (Fig. 3) also revealed that slavemaker samples clearly cluster first by species and secondarily by raiding phenotype. However, while hosts also cluster primarily by species, secondary clustering by phenotype is far less apparent.

**Figure 2:**
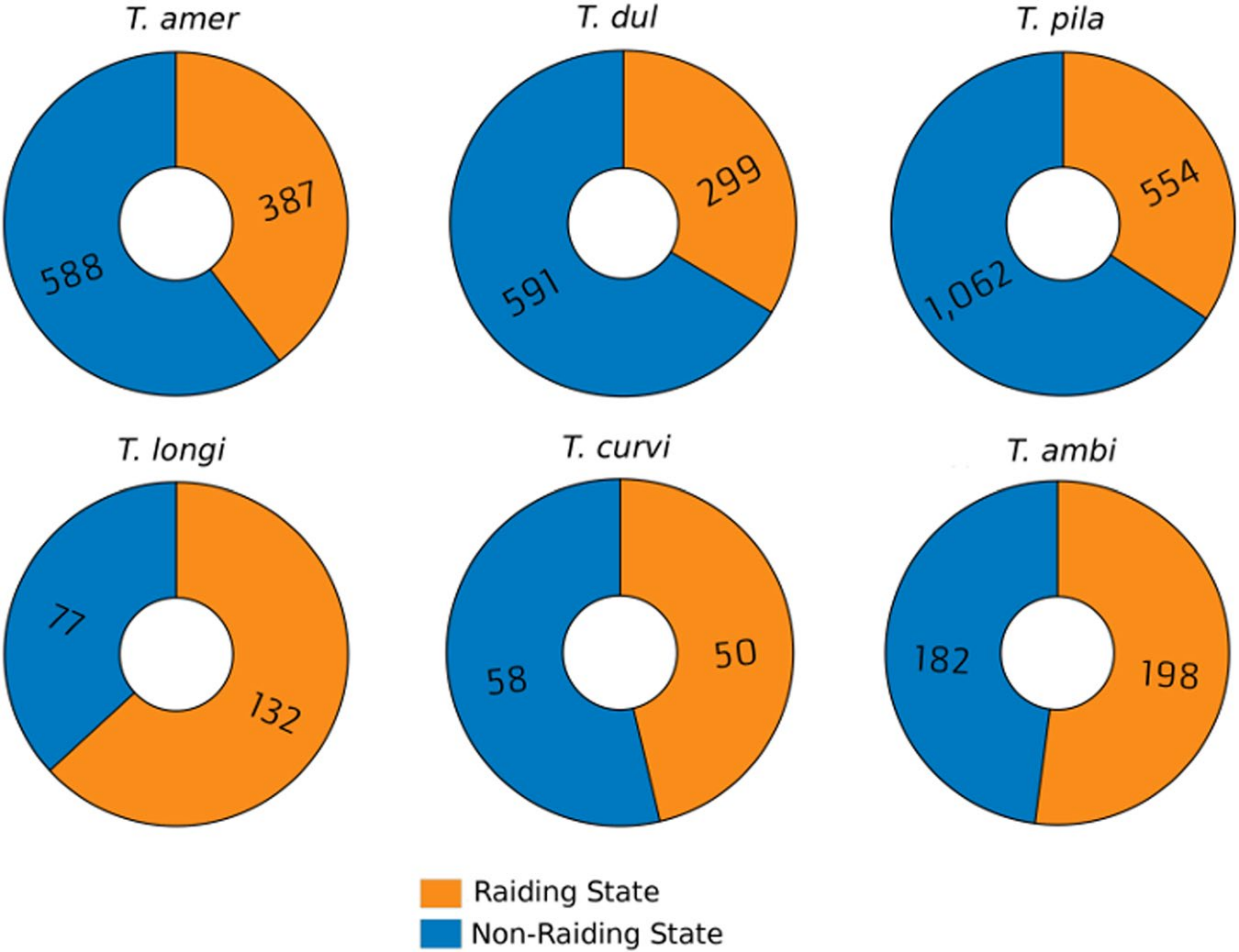
Number of genes found to be differentially-expressed within six *Temnothorax* species, up-regulated during either slavemaker or host raiding or non-raiding behavioral states. Upper row: Slavemakers; Bottom row: Hosts. Species abbreviations are as follows: *T. ambi: T. ambiguus, T. curvi: T. curvispinosus, T. longi: T. longispinosus, T. amer: T. americanus, T. dul: T. duloticus, T. pila: T. pilagens*.

**Figure 3:**
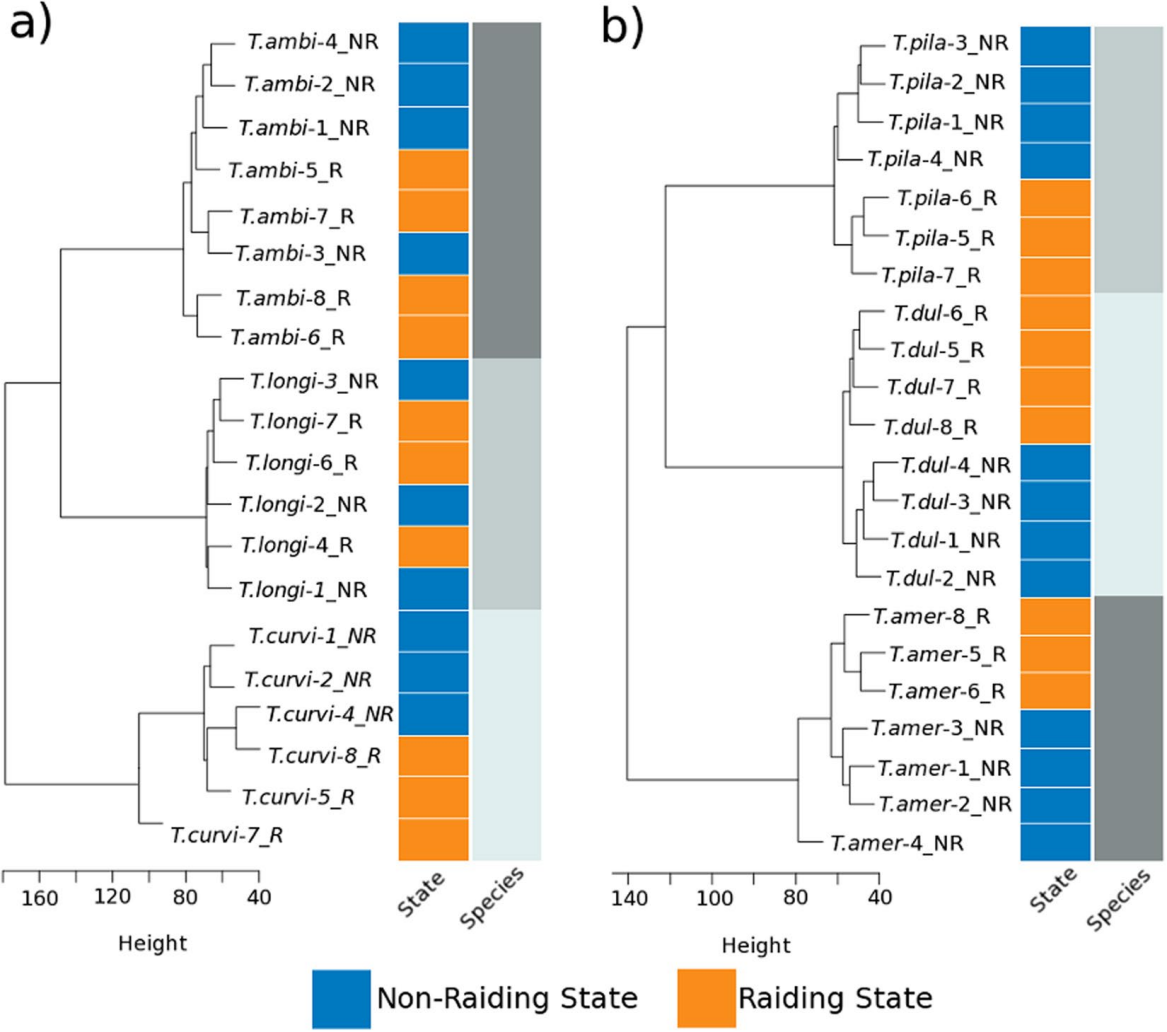
Dendrograms resulting from WGCNA of orthologous clusters. (**a**) WGCNA of host-specific clusters shows that samples group primarily according to species. Phenotype does not appear to have a strong influence on host grouping. (**b**) WGCNA of slavemaker-specific clusters yields patterns of grouping driven first by species and then secondarily by phenotype. Unlike hosts, phenotype does appear to be associated with more similar gene expression patterns within slavemakers.

**Figure 4:**
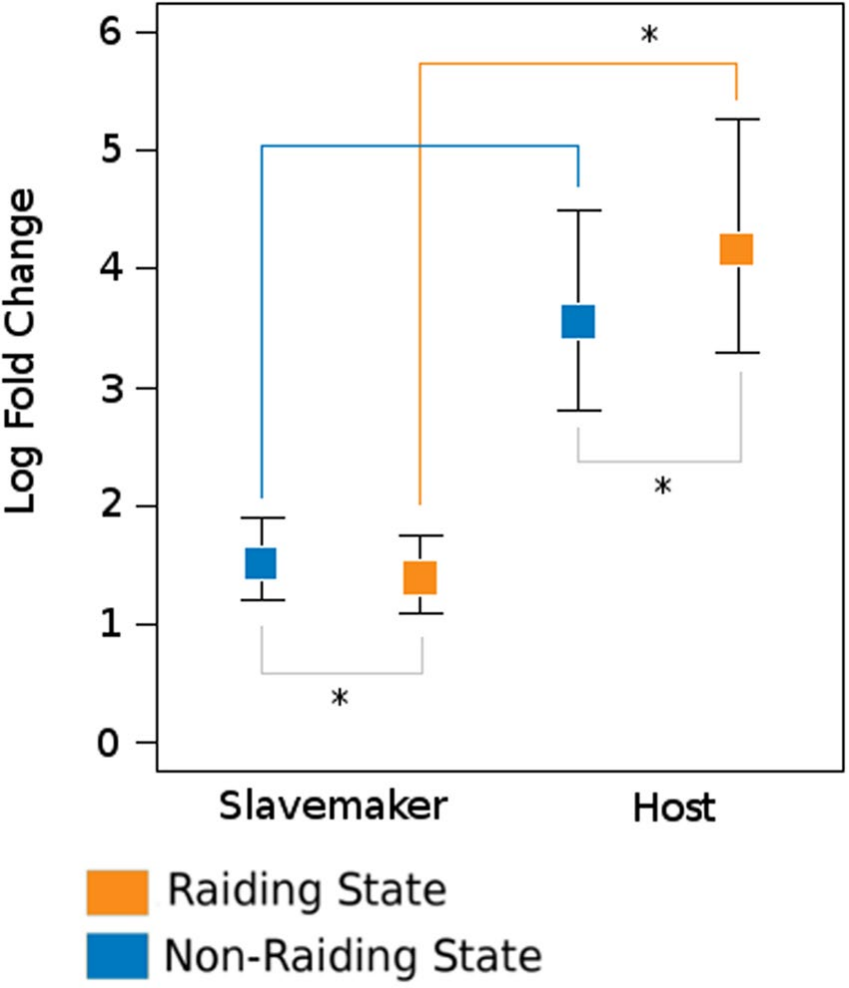
Average log fold change of differentially-expressed genes between behavioural phenotypes of hosts and slavemakers herein examined. While non-raiding phenotypes only show a trend towards differentiation between slavemaker and host (p < 0.059), the log fold change in expression between slavemaker and host during the raiding/defensive phenotypes (p < 0.028) does differ significantly. Log fold change within lifestyle groups between raiding and non-raiding phenotypes differ significantly for both slavemakers (p < 0.013), as well as hosts (p < 0.028).

Providing additional insight into the differences in expression patterns between slavemaker and host, we also find that while slavemakers had in total more genes differentially expressed, the log fold change of DEGs was *lower* in slavemakers when compared to hosts (lmer: lifestyle: χ² = 9.14; p < 0.0025; behaviour: χ² = 1.69; p = 0.19; lifestyle *x* behaviour, χ² = 9.44; p < 0.0022; Fig. 4). Moreover, in slavemakers, the many up-regulated genes during the raiding phenotype shifted their expression *less* than up-regulated genes in the non-raiding phenotype, whereas the opposite holds true for the hosts (Fig. 4).

**Figure 5:**
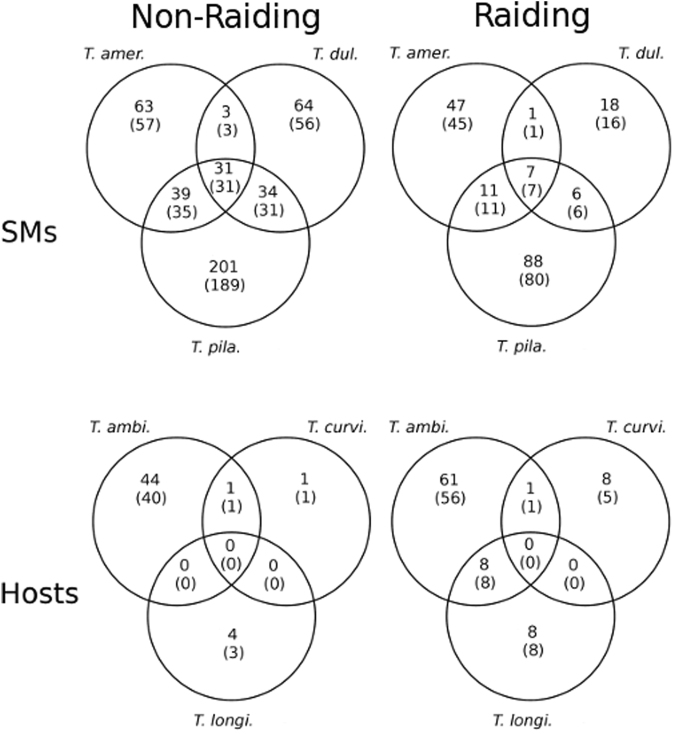
Venn diagrams displaying the number of significantly differentially-expressed contigs that are either private to a specific species, or shared by multiple species. Homology determined by cluster analysis. Un-bracketed numbers indicate total number of clusters, bracketed numbers indicate subset of clusters with accompanying functional annotation. For species abbreviations, see Fig. 1.

In order to determine which genes shared similar expression patterns between species, we utilized gene cluster orthologs produced during a sister study^19^. The resulting Venn diagrams (Fig. 5) allowed us to visualize the number of genes possessing species-specific or shared expression patterns between species and phenotype. Within both slavemaker and host non-raiding phenotypes, the proportion of genes a] sharing expression patterns within at least two species (χ² = 8.037; p = 0.005), and b] possessing species-specific patterns of expression (χ² = 13.230; p < 0.0001), was higher in slavemakers. We found no difference in the ratio of commonly to privately expressed genes between slavemakers and hosts during their respective raiding phenotypes (χ² = 0.662; p = 0.416), though between non-raiding phenotypes, slavemakers show a trend towards a higher proportion of genes up-regulated compared to hosts (χ² = 3.807; p = 0.0511). Additionally, during the raiding phenotypes of all species examined, we note no significant difference in the ratio of genes commonly up-regulated (χ² = 3.47; p = 0.062), up-regulated by only two species (χ² = 0.02; p = 0.881), or privately up-regulated (χ² = 0.662; p = 0.416), between lifestyles. A list of DEGs and their expression stats may be found in Supplementary Table S4.

**Figure 6:**
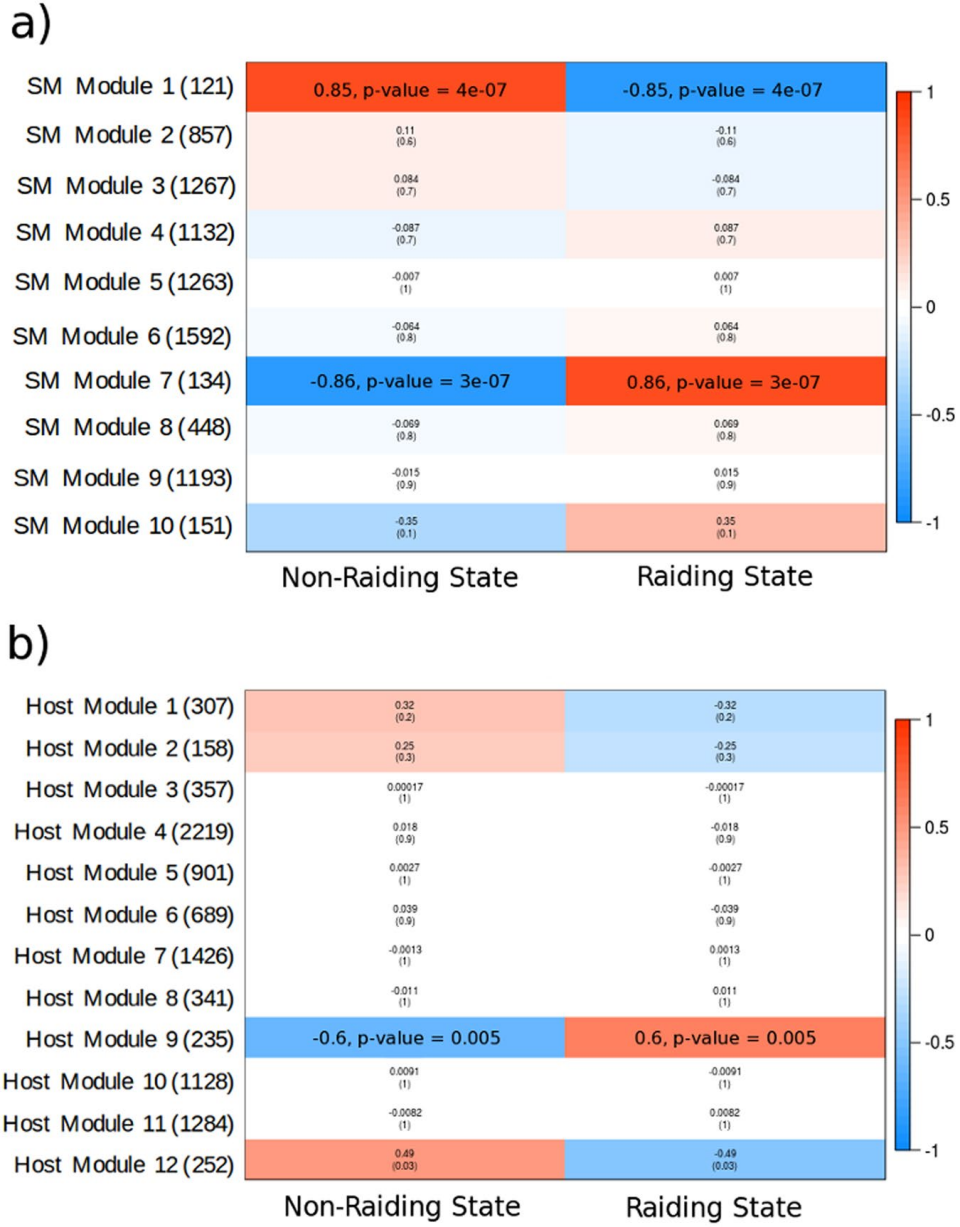
Module-Trait relationships within all three slavemaking species (**a**) and all three host species (**b**). Contigs within Slavemaker Module 1 are strongly associated with the non-raiding phenotype, where contigs in slavemaker Module 7 are strongly associated with the raiding phenotype. Host contigs within Host Module 9 are positively associated with the defensive phenotype. Numbers to the right of module identifiers indicates the number of contigs within that specific module. Slavemaker Module 10 and Host Module 12 are “leftover” modules, containing random contigs that did not fall into any other module.

WGCNA across all three slavemaking species revealed that one group of contigs with similar expression patterns across behaviors (gene module) is significantly positively associated with the raiding phenotype and one module significantly positively associated with the non-raiding phenotype (Fig. 6; Supplementary Fig. S4). Functional enrichment of contigs within each module reveal that Slavemaker-Module-1 shows a significant bias towards translation and various metabolic functionalities (Supplementary Table S5), while contigs in Slavemaker-Module-7 show a significant functional enrichment of translation, response to oxidative stress, and various additional metabolic functions (Supplementary Table S6). Hosts only show one significantly-enriched module, here positively associated with the raiding phenotype (Fig. 6b). Functional enrichment of this module (Host-Module-9) reveals that lipid metabolic and isoprenoid biosynthetic processes, among others, are over-represented within these contigs (Supplementary Table S7). Unsurprisingly, given that slavemaker raids and host nest defense show few external behavioural similarities, we find no modules with significant patterns of shared expression when performing WGCNA upon all six *Temnothorax* species herein examined (Supplementary Fig. S5).

**Figure 7:**
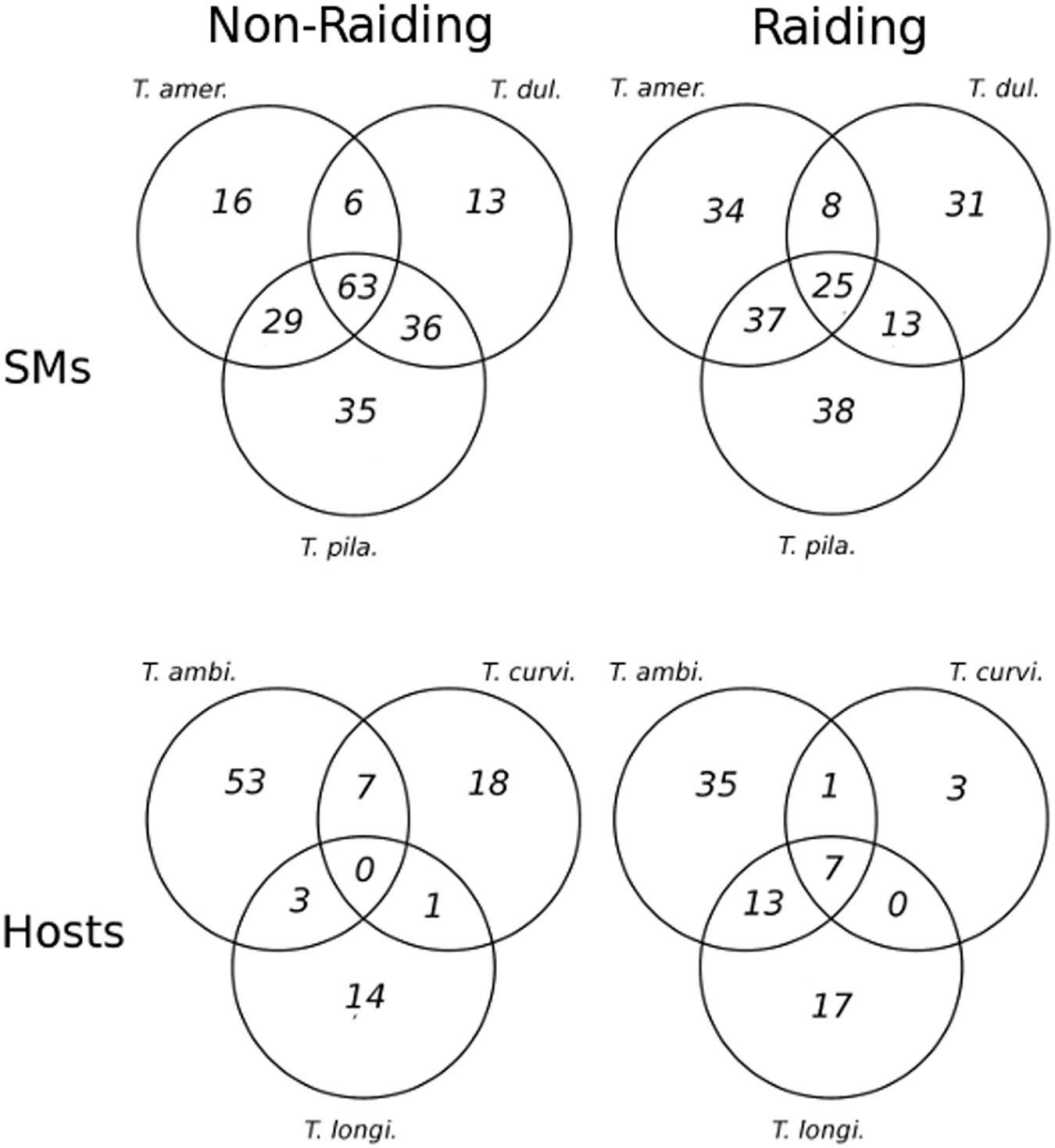
Venn diagrams displaying the number of KEGG pathways that are either private to a specific species, or shared by multiple species. Pathways generated by obtaining KO (KEGG Pathway) terms for phenotype-specific, significantly-differentially-expressed genes. For species abbreviations, see Fig. 1.

Additionally, comparative pathway analysis reveals that slavemakers display a greater number of commonly over-represented pathways relative to privately over-represented pathways during both raiding (χ² = 6.5; p = 0.011) and non-raiding phenotypes (χ² = 81.8; p < 0.0001) compared to hosts (Fig. 7). Within slavemakers, fewer pathways shared over-representation across species in the non-raiding phenotype than the raiding phenotype (χ² = 20.8; p < 0.0001); whereas the reverse is true for the hosts, in which more pathways were commonly up-regulated during the raiding than the non-raiding phenotype (χ² = 7.3; p < 0.007).

### Functional Enrichment

Examination of functional enrichment results, performed on (1) groups of genes with species-specific expression patterns within a single phenotype, (2) groups of genes with expression patterns shared between two or more species within a single phenotype, and (3) all genes differentially expressed by a single species in a specific phenotype, revealed a number of functions over-represented both within and between species (Supplementary Figs S6–S21). While host species did not possess enough enriched functions for additional comparison, we were able to further analyze those functions found to be enriched within slavemaking species both in their non-raiding (Supplementary Fig. S22) and raiding (Supplementary Fig. S23) phenotypes. Far more functions appear within all examined slavemaking species in their non-raiding phenotype. Indeed, the entire enriched functional repertoire of *T. duloticus* in its non-raiding phenotype is shared by *T. americanus* and *T. pilagens* (Supplementary Fig. S22). Contrasting this, the raiding phenotype is characterized largely by the enrichment of species-specific functions and processes, with no enriched functions being shared between all three slavemaking species (Supplementary Fig. S23). GO terms of enriched functions within Supplementary Figs S22 and S23 may be found in Supplementary Table S8.

## Discussion

Guided by three primary assumptions about the molecular evolution of social parasitism: (1) trait novelty or commonality will be reflected at the molecular level; (2) Novel molecular processes underlie lineage-specific phenotypes; and (3) that conserved regulatory processes underlie the response to a shared environment^34^, this study utilized an RNA-Seq approach following behavioural experiments in order to identify regulatory patterns involved in slavemaker raiding behaviour and host defensive behaviour by focusing on the three *Temnothorax* slavemakers and their three primary host species (Fig. 1). Indeed, by comparing multiple slavemaker and host species, we can determine whether or not raiding and defensive phenotypes evolved along independent, species-specific trajectories, or if these phenotypes arose in parallel within *Temnothorax*.

### Expression Analysis

In keeping with our first prediction - that trait novelty or commonality will be reflected at the molecular level – we were able to detect a number of regulatory differences between species and lifestyles within this transcriptome study. As expected, we find more genes differentially expressed between raiding and non-raiding phenotypes in slavemakers than within hosts. Moreover, slavemakers seem to down-regulate a relatively large number of genes during raiding, suggesting that slavemakers focus their gene expression for the singular and crucial task of raiding. Together, these patterns are likely driven by the highly dissimilar physiological and behavioural phenotypes of slavemakers out of raiding season and hosts before nest defense. Slavemakers switch from their inactive state in spring to a highly active state for a few weeks in summer. Hosts, however, become active after winter and carry out their normal daily chores until they are attacked by slavemakers, which then necessitates a rapid, though short-term, response. Thus, with regard to the disparity in raw number of DEGs between lifestyles, it is likely that we are observing an equipoised gene expression pattern - in conjunction with some short-term changes - within slavemaking species during raiding. This is in direct contrast to host species, where we observe a radical short-term shift in expression of a few genes as host defenders have only minutes or seconds to respond to a slavemaker attack. However, due to differences in physiology between raiding and stay-at-home slavemakers, it must be noted here that the time of sampling for the non-raiding phenotype between host and slavemaker was about two months apart (see Material and Methods for further explanation). Still, taken together, these patterns suggest that gene regulation is fundamentally different between slavemaking and host species within these phenotypes; a finding that is corroborated by accompanying study investigating genes under selection within these same six species^19^.

Additionally, we find largely species-specific patterns of expression for most DEGs (Fig. 4), suggesting that, while all three slavemakers conduct raids, and all host species defend their colonies against such raiding attempts, these responses are largely controlled via unique, species-specific mechanisms. Within slavemakers, this pattern is in keeping with our second prediction that novel molecular processes underlie lineage-specific phenotypes, where the more varied slavemaker raiding phenotypes^22,24,46,59^ are reflected in a greater number of genes under differential regulation between species. By comparison, the relatively low number of DEGs within host species is striking though not unexpected, as this finding is in keeping with our final prediction - that conserved regulatory processes underlie the response to a shared environment and similar evolutionary pressures - and is likely the result of overall mechanistic similarity, and the short transition time, between these two phenotypes in host species. Additionally, hosts share no overlap in those genes that were determined to be differentially expressed between raiding and non-raiding phenotypes, indicating that hosts utilize comparatively minor, species-specific regulatory shifts that correspond with ecological pressure exerted by local social parasites^41,42,60,61^.

While host species did not possess enough DEGs for effective functional enrichment, functional enrichment analysis of slavemaking species did yield additional insight into the similarity, or lack thereof, of biological processes underlying slavemaker behaviour and physiology during raids as well as out of raiding season. When out of raiding season, slavemakers share a large proportion of enriched functions, with the entire functional repertoire of *T. duloticus* mirrored in both *T. americanus* and *T*. *pilagens* (Supplementary Fig. S22). Contrast these findings with the shared proportion of enriched functions between slavemaking species during raiding (Supplementary Fig. S23), where we find much less similarity between species. Taken together, these findings strengthen our initial assertion that, while the non-raiding phenotype of slavemakers is characterized by a degree of molecular - and subsequently, functional - commonality, raiding phenotypes are regulated in a species-specific manner.

### Genes of Interest

One frequently recurring gene, found up-regulated during raiding within all three slavemaker species is *Acyl-CoA-Delta (11) desaturase* (Table 1)*;* a gene found previously to be involved in pheromone biosynthesis^62^. Given that *Temnothorax* slavemakers employ a number of subversive chemical weapons – from Dufour’s gland secretions that elicit fighting among host defenders^60^, to CHC profile modifications during raiding^27,29^ – we speculate that regulatory shifts of esterase and desaturase genes^62^ involved in these mechanisms could impart a number of benefits to raiders: from desiccation resistance during raiding activity out of their own nest to CHC-masking, making chemical detection of slavemaker raiders by host defenders less likely^24,29^. However, that *Acyl-CoA-Delta (11) desaturase* was also found to be up-regulated in *T. ambiguus* during its raiding behavioral state is unusual. This pattern of expression across lifestyles does seem to further suggest that *Acyl-CoA-Delta (11) desaturase* is involved in CHC production in *Temnothorax* ants – and not chemical weapons for raiding – as there is no evidence that *T. ambiguus* uses chemical weapons against raiding slavemakers.

**Table 1:** Expression statistics for genes of interest. Positive log fold change indicates gene up-regulation during the raiding phenotype, negative log fold change indicates up-regulation during non-raiding phenotype. Species abbreviations are as follows: *T. ambi: T. ambiguus, T. curvi: T. curvispinosus, T. longi: T. longispinosus, T. amer: T. americanus, T. dul: T. duloticus, T. pila: T. pilagens*. Note: a single gene can occur multiple times for a single species. *De novo* assembly of the transcriptome may lead to multiple contigs per gene due to low coverage of certain regions, or due to splice variants.

Gene	Species	Up-Regulated	LogFC	FDR-Value
*Acyl-CoA-Delta (11)*	*T. amer*.	Raiding State	0.90	<0.01
*desaturase*		Raiding State	2.03	<0.01
	*T. dul*.	Raiding State	6.20	<0.01
		Raiding State	0.65	0.01
	*T. pila*.	Non-Raiding State	−1.57	<0.01
		Non-Raiding State	−1.38	<0.01
		Raiding State	0.49	0.04
		Raiding State	0.78	<0.01
	*T. ambi*.	Raiding State	3.14	<0.01
*Vitellogenin-6*	*T. dul*.	Raiding State	0.93	0.02
	*T. pila*.	Raiding State	2.06	0.01
	*T. longi*.	Raiding State	2.10	<0.01
		Raiding State	2.12	<0.01
*Vitellogenin-3*	*T. dul*.	Non-Raiding State	−2.40	0.01
	*T. ambi*.	Raiding State	3.47	0.01
*Vitellogenin*	*T. dul*.	Non-Raiding State	−1.94	<0.01
*Receptor*		Non-Raiding State	−2.34	<0.01
		Non-Raiding State	−2.20	<0.01
	*T. pila*.	Non-Raiding State	−1.06	0.05
	*T. ambi*.	Raiding State	2.48	<0.01
*Trypsin-7*	*T. amer*.	Non-Raiding State	−11.07	<0.01
		Non-Raiding State	−9.82	0.02
	*T. dul*.	Non-Raiding State	−6.71	<0.01
		Non-Raiding State	−7.60	<0.01
	*T. pila*.	Non-Raiding State	−6.85	<0.01
		Non-Raiding State	−6.39	0.04
	*T. ambi*.	Raiding State	5.94	0.04
		Raiding State	8.13	<0.01
		Raiding State	8.26	<0.01
	*T. curvi*.	Raiding State	9.18	0.01
	*T. longi*.	Raiding State	5.69	<0.01
		Raiding State	8.47	0.03
		Raiding State	8.90	<0.01
		Raiding State	9.30	0.01
		Raiding State	9.54	0.01
		Raiding State	9.92	0.01
*Trypsin Inhibitor*	*T. pila*.	Raiding State	1.52	<0.01
*Kelch*	*T. pila*.	Non-Raiding State	−4.40	0.01
	*T. longi*.	Non-Raiding State	−6.16	0.05
*painless*	*T. amer*.	Raiding State	1.84	<0.01
	*T. curvi*.	Raiding State	10.45	0.04
*MYG1*	*T. amer*.	Non-Raiding State	−1.63	<0.01
	*T. curvi*.	Non-Raiding State	−8.29	0.04

*Trypsin-7* was also found to possess a particularly interesting pattern of expression: it was universally down-regulated in slavemaking species during raiding while, conversely, was up-regulated universally in hosts during raiding (Table 1). A previous study into the function of *Trypsin-7* revealed its role in digestion and, potentially, host-seeking behaviour within the malaria mosquito *Anopheles gambiae*^63^. Given the expression data produced here, *Trypsin-7* expression certainly does not appear to be positively correlated with host-seeking as in *A. gambiae*. That *Trypsin-7* expression is strongly negatively correlated with the slavemaker raiding phenotype does seem to suggest that *Trypsin-7* is somehow involved in the control of this behavior. Indeed, even within the context of raiding, *Tyrpsin-7* might retain some of its digestion functionality - as food restriction does trigger an increase in raiding activity in *T. americanus*^64^; though determining the exact mechanisms involved are beyond the scope of this study. However, given the sampling method of this experiment, it is difficult to disentangle raiding phenotype effects from seasonal or unrelated physiological effects. That an unspecified *Trypsin Inhibitor* was found to be strongly up-regulated during raiding behaviour within *T. pilagens* might indirectly shed some light onto the hypothetical role of *Trypsin-7*. Assuming that *Trypsin-7* prevents slavemaker raiding behaviour, we postulate that this *Trypsin Inhibitor* is at least involved in the suppression of *Trypsin-7*, in turn facilitating raiding behaviour within *T. pilagens*. Again, however, additional gene-specific approaches are required in order to elucidate the importance of *Trypsin Inhibitor* to the raiding phenotype of *T. pilagens*, as well as the precise interplay between *Trypsin Inhibitor* and *Trypsin-7*.

While the exact function of specific genes cannot be determined within the purview of this study – indeed functional verification of many genes found here could be accomplished through RNA-mediated gene knockdown – the proposed functions of DEGs is nonetheless insightful into the potential processes and mechanisms that define species-specific phenotypes, or are maintained within like phenotypes.

## Conclusions

While it has long been known that slavemakers display lineage-specific chemical and behavioural phenotypes during raids^22,24,46,59^, here we provide the first evidence for the underlying gene expression patterns governing the raiding phenotype within *Temnothorax* slavemaking ants; as evidenced by the comparatively high number of orthologous genes found to possess species-specific patterns of expression. That this same pattern is observed on the pathway levels as well suggests regulatory and, ultimately, functional divergence of molecular mechanisms underlying the raiding phenotype in *Temnothorax* slavemakers. Not unexpectedly, slavemaking species display a much higher level of regulatory similarity when out of raiding season, where these species are universally inactive and do not engage in normal nest tasks. A similar pattern was also observed in workerless parasite species within the genera *Pogonomyrmex* and *Vollenhovia*^65^. Despite being of different genera, the behavioral differences between these species is not due to sequence change or gene loss, but attributed to differential expression patterns of gene sets. Our results seem to reflect this finding, as behavioral diversification among slavemakers and hosts in *Temnothorax* appear to be typified by species-specific patterns of gene expression; however the extent of gene loss and sequence change, and the precise importance of these mechanisms within the context of *Temnothorax* slavemakers, is beyond the purview of this investigation.

Additionally, we note that hosts possess largely species-specific molecular responses to slavemaker aggression – which are driven by comparatively small shifts in regulatory mechanisms – also suggesting regulatory differences in orthologous genes as well as species-specificity at the pathway level between host species during nest defense.

Functionally, we find a diverse repertoire of DEGs both within slavemakers as well as in hosts. Broad characterization of slavemaker raiding behaviour includes the universal up-regulation of *Acyl-CoA Delta (11) desaturase* genes, which are likely involved in the production of olfactory signals in slavemakers or modification of the cuticular hydrocarbon profile in both slavemakers and hosts. While the raiding strategies of all slavemaking species examined here differ substantially from one another, all rely on an altered chemical secretion to enhance the chance of raiding success^31^. Perhaps most interestingly are the differential expression of *Trypsin-7* and its suspected controller *Trypsin Inhibitor*. Further analysis of genes found here to be significantly differentially-expressed - likely through the use of RNA-mediated gene knock-down (RNAi), followed by extensive behavioural analysis - is necessary in order to more clearly elucidate the exact role of these genes within the context of slavemaker raiding and host nest defense behaviour. Taken together, and framed by previous studies^34,65^, our findings suggest that the evolution of molecular mechanisms underlying slavemaker raiding and host nest defense phenotypes in *Temnothorax* is characterized by high flexibility and lineage-specificity.

## Material and Methods

### Sample Collection and Raiding Experiment

Ant colonies were collected in spring 2012 and 2013 at three locales in the Northeastern US (Supplementary Table S9) and transported in Ziploc bags within their natural nest sites. Upon arrival to the laboratory at the Johannes Gutenberg University in Mainz, each colony was transferred into its own plaster-floored nesting box, containing a single slide-nest into which the colony relocated. A slide nest is an artificial nesting site comprised of a small Plexiglas cavity sandwiched between two glass microscope slides. Colonies were then kept under a constant 20 °C, 14 L:10D light cycle and were fed twice weekly with honey and crickets. All colonies used in raiding experiments were transferred to 25 °C, 14 L:10D light cycle conditions one week prior to the onset of the raiding experiment in order to promote an increase in scouting and raiding activity in slavemakers.

Each slavemaker species was allowed to raid colonies of its preferred host species from the same community. Raids using colonies of *T. americanus* vs *T. longispinosus* from the New York site, and *T. duloticus* vs. *T. curvispinosus* from the Ohio site were conducted in 2012, whereas raids involving *T. pilagens* vs. *T. ambiguus* from Michigan were conducted in 2013. All 36 raids, i.e. 12 per host-slavemaker pair, were performed in the year of collection. On each day of the raiding experiments, five raiding arenas were set up in the laboratory, into which a host and a slavemaker colony were placed. This setup gave slavemakers the opportunity to raid a host nest. If a slavemaker performed no raid on a specific day, both host and slavemaker colonies were placed back in their respective nest boxes overnight, and the experimental procedure repeated again on the following day until a successful raid occurred (for a maximum of 14 days). On the first day of each raiding experiment, two foragers (outside individuals) were removed per host colony as “before raid” individuals for later transcriptome analyses. Then one host colony and one slavemaker colony (residing in their slide-nest) were transferred to diagonally-opposite corners of a 30 × 40 cm plastic box with plastered floor. The plaster was kept moist throughout the experiment to prevent desiccation. Within this raiding chamber, opposite the slavemaker colony, honey and water was provided. Colonies were observed continuously until the onset of a raid. We waited until slavemaker scouts had returned to their mother nest and recruited additional raiders in order to infiltrate the host nest, and aggressive encounters could be observed between slavemaker and host workers. At this point, two slavemaker and two host workers per colony, directly engaged in aggressive interactions just outside of the artificial nest, were collected. As slavemaker workers show raiding activity - i.e. leave the colony in search for host nests throughout the raiding season from July to September - we decided to wait until mid-October to collect “out of raiding season” slavemakers. Two weeks before sampling in fall, colonies were again moved to 25 °C, 14 L:10D light cycle conditions, so that environmental conditions were the same as during the raiding experiments in summer. Only infertile slavemakers engage in raiding activity, whereas fertile slavemaker workers remain in the nest during the raiding season^64,66^, so that we dissected the ovaries of slavemakers to select infertile workers during both sampling points. Laboratory-based raiding experiments were deemed acceptable given the inherent difficulty – due largely to the small size of individuals and low number of workers involved in raiding parties – in facilitating and observing raids within the field. Indeed, even in the field, the foraging and raiding ranges of *Temnothorax* tends to be short^67^. Additionally, living and laboratory conditions could be standardized for all colonies throughout the experiment.

Unfortunately, the intrinsic nature of our system does not allow for a “clean” experimental set up. Raiding versus non-raiding slavemakers could have been sampled in two different ways: either by comparing younger, fertile stay-at-home slavemakers with older, infertile raiding slavemakers; or by comparing infertile raiders to the same behavioural caste outside of the raiding season. Both approaches introduce method-specific confounding factors: fertility and age using the first approach, and physiological makeup of individuals due to seasonal differences using the latter. The latter method was ultimately chosen, as we expected that seasonal changes would influence gene expression far less strongly than fertility and age - especially when external conditions, such as temperature and humidity, are kept the same for all colonies. As hosts alter their behaviour in the long term after a slavemaker encounter, generally in the form of persistent elevated aggression^42,68^, we decided to sample host workers on the day of the raids, just before slavemaker contact, as well as during raids. All workers collected during this experiment for later transcriptome analysis were transferred directly into 500 µl Trizol (Invitrogen) and homogenized before freezing and stored at −80 °C. Ants were sampled at different times of day; since it is impossible to plan or instigate a raid, individuals must be collected whenever a raid takes place, independent of time of day. Additionally, daytime variation between individuals should be canceled out by our pooling strategy (see below).

### RNA Isolation and Sequencing

As we are less interested in individual differences in gene expression, but in general changes associated with specific behavioural *and* physiological states, we pooled whole bodies of six individuals per replicate (two individuals from three colonies each)^57^. We pooled the same colonies across treatments to keep possible colony-dependent variation constant across treatments. In total, four samples per species per behavioural phenotype were generated, resulting in a total of 48 samples. While pooling does indeed result in a loss of data at the individual level, this elimination of individual variation also strengthens common signals^57^. For example, here, since time of day (of collection) cannot be controlled for due to the unpredictable nature of raiding, pooling individuals of the same colony mitigates variation due to collection time. Additionally, physiological factors can influence behaviour in insects^69,70^. As such, focusing our RNA-Seq approach entirely on brains, for example, excludes all physiology-associated molecular signals, which might have important down-stream affects upon behaviour.

For RNA isolation, we followed the protocol of the Center for Genomics and Bioinformatics Bloomington (https://dgrc.bio.indiana.edu/include/file/CGB-TR-200610.pdf). In short, 200 µl chloroform was added to each Trizol sample and the mixture shaken vigorously for 15 seconds, and then centrifuged for 15 min at 4 °C and 11,000xg. The upper aqueous phase was transferred to a new 1.5 ml RNase-free tube and precipitated with 200 µl of absolute ethanol. The solution was gently pipetted four times and transferred to an RNeasy mini-spin column (Qiagen). Further procedure followed step three onwards of the RNeasy Clean-Up manual (Qiagen). Illumina library preparation with individually marked (MID) libraries was performed through GENterprise Genomics, affiliated with Mainz University (http://www.genterprise.de/), and paired-end sequenced on an Illumina HiSeq. 2000. Raw reads were analyzed for quality using FastQC v0.11.2, and Illumina adapters cut from all sequences with Trimmomatic v0.32^71^ using the following parameters: 2:30:10:8:TRUE LEADING:3 TRAILING:3 SLIDINGWINDOW:4:20 MINLEN:20 HEADCROP:13. Reads may be obtained from NCBI short read archive (Accession Number GSE95604).

### *De novo* transcriptome assembly

After analyzing the quality of our initial data, as obtained from the sequencing facility, we tested a number of *de novo* assembly and analysis approaches to determine which method yielded the best-assembled transcriptomes. Assembly methods examined included standard, short-read assembly approaches using Trinity^72^ and CLC Workbench v.7.0.3 (https://www.qiagenbioinformatics.com/), followed by a meta-assembly approach using EvidentialGene (https://sourceforge.net/projects/evidentialgene/) and MIRA^73^. For meta-assembly of the transcriptome, a same-mixed pattern of replicate-matching was utilized (BeforeRaid1-BeforeRaid2, BeforeRaid3-DuringRaid3, DuringRaid1-DuringRaid2, BeforeRaid4-DuringRaid4) for the CLC Workbench phase of the assembly. Using all reads at once decreased assembly quality, and we therefore decided for a step-wise assembly approach. Default parameters were used for these CLC assemblies except for ‘bubble size’ – auto, and a ‘word size’ of 35. Final transcriptome assembly was performed with MIRA, using CLC Workbench contigs as input. Trinity and EvidentialGene assemblies were performed using default settings. Transcriptome assembly was followed by the removal of redundant and/or low-confidence contigs from each transcriptome using CD-Hit-Est v.4.6.1^74^.

After the successful assembly of all transcriptomes, we looked at a number of factors to assess assembly quality including (1) total number of contigs, (2) average contig length, (3) percent coverage, and (4) contig BLAST hit rate. Qualimap v.2.1^75^ was used to view the.bam output from TopHat v.2.0.13^76^, as well as determine the average number of raw read hits of each base within a contig. Based on the above-mentioned analyses we decided to use the CLC + MIRA meta-assembly for the following analyses. Summary statistics for each assembly can be viewed in Supplementary Table S1.

### Differential Gene Expression Analyses

In order to determine which genes were most active during a specific behaviour, we utilized a gene expression approach in which we compared expression levels within a single species, between the two behavioural phenotypes. To accomplish this, we used EdgeR v3.9.12^77^, an add-on package for R v.3 (R Core Team, 2015). TopHat v2.0.13^76^, in conjunction with Bowtie 2 v2.1.0 (http://bowtie-bio.sourceforge.net/) were used to align reads to their corresponding contigs. eXpress v1.5.1 (http://bio.math.berkeley.edu/eXpress/) was used to obtain read count information. After initial expression analyses, PCA and NMDS analyses were performed in R using ‘vegan’ and ‘MASS’ libraries to determine whether samples grouped primarily by species or by phenotype (Supplementary Fig. S1), and how well samples grouped within a species (Supplementary Figs S2 and S3). While all gene expression analyses are based upon all species-specific contigs, between-species comparisons are based upon lifestyle (slavemaker or host)-specific orthologous clusters. These ortholog sequence clusters were constructed using OrthoMCL 2.0.9^78^ during a sister study^19^. Thus, to elucidate which differentially-expressed genes (DEGs) were shared between species, we utilized these orthologous clusters, matched with previously-determined DEGs, in combination with Venny v2.1.0 (http://bioinfogp.cnb.csic.es/tools/venny/). Next, we elucidated the broad functionality of DEGs through both functional analysis as well as metabolic pathway mapping. Contigs within all assemblies were functionally annotated, using NCBI’s BlastX v.2.2.30 against NCBI’s November 2014 non-redundant arthropod database. Functional enrichment analyses were performed using the Enrichment Analysis (f-test) functionality of Blast2GO Pro v3.2/3.3 (https://www.blast2go.com/) with default settings. We utilized the KEGG Automatic Annotation Server (KAAS) to assign KEGG Orthology (KO) terms to contigs^79^. Acquisition of KO terms for contigs was followed by the use of KEGG Mapper (Reconstruct Pathway) to obtain pathways associated with each KO term (http://www.genome.jp/kegg/tool/map_pathway.html).

The number of differently expressed genes and pathways between species were compared using chi-square tests. Differences in log fold change of DEGs were analyzed with a general linear-mixed model (lmer function implemented in the lme4 package^80^). All statistical analyses were conducted in R v. 3.3.1. Finally, weighted gene co-expression network analysis (WGCNA) was performed in R using package WGCNA^81^, followed by Kruskal-Wallis tests using package ggpubr in order to determine how the expression of groups of contigs is associated with shifts in phenotype. Initial clustering for the production of dendrograms was carried out using the WGCNA sub-function Hclust with default parameters.

### Data Accessibility and Additional Information

Raw Reads deposited into NCBI’s GEO database under Accession Number GSE95604.

## Electronic supplementary material

Supplementary Information

Supplementary Dataset 2

Supplementary Dataset 3

Supplementary Dataset 4

Supplementary Dataset 5

Supplementary Dataset 6

Supplementary Dataset 7

Supplementary Dataset 8
