# Supplementary material for: Comparative analyses of co-evolving host-parasite associations reveal unique gene expression patterns underlying slavemaker raiding and host defensive phenotypes
Source: Sci Rep. 2018 Jan 31;8:1951. doi: 10.1038/s41598-018-20262-y (PMC5792630; doi:10.1038/s41598-018-20262-y)
Supplement: Supplementary file 1 — Supplementary Information [file 41598_2018_20262_MOESM1_ESM.doc]

**Comparative analyses of co-evolving host-parasite associations reveal unique gene expression patterns underlying slavemaker raiding and host defensive phenotypes**

Austin Alleman^1^, Barbara Feldmeyer²*, Susanne Foitzik^1^*

* Authors contributed equally to the study.

^1^*Institute of Organismic and Molecular Evolution, Johannes Gutenberg University Mainz, Johannes von Müller Weg 6, Mainz 55128, Germany*

*²Senckenberg Biodiversity and Climate Research Centre, Senckenberg Gesellschaft für Naturforschung, Senckenberganlage 25, D-60325 Frankfurt am Main, Germany*

**Corresponding author**: Austin Alleman, email: aalleman@uni-mainz.de

Supplementary Material and Methods:

*Supplementary Results*

**Transcriptome Sequencing and Assembly** A number of assembly methods were tested - 1] CLC + MIRA meta-assembly with no post-transcriptome refinement, 2] CLC + MIRA meta-assembly with refinement using CD-Hit, 3] CLC + MIRA meta-assembly with refinement using both CD-Hit and Qualimap, 4] Trinity assembly without refinement, 5] Trinity assembly with refinement with CH-Hit, 6] Trinity with refinement using Qualimap, and 7] EvidentialGene assembly - in order to determine which transcriptome assembly method yielded the most complete results for our analysis. Here, we found that EvidentialGene, on average, yielded the longest contig sequences, but the lowest percentage of coverage, according to TopHat (Supp. Table S43). Transcriptomes produced via CLC + MIRA meta-assembly possessed contigs nearly the same length as those produced by EvidentialGene, but with a much higher percentage of coverage. For our data, Trinity, regardless of refinement, yielded higher coverage than EvidentialGene, but a far greater number of short contigs. From this, we conclude that CLC + MIRA and EvidentialGene yield more complete contigs, less prone to gene fragmentation when compared to the Trinity assemblies. However, while EvidentialGene did produce the longest contigs, the percent coverage is relatively low. Thus, in light of these observations, we decided to base further analyses upon those transcriptomes obtained from CLC + MIRA meta-assembly with refinement using both CD-Hit and Qualimap.

Finalized transcriptomes from all six examined species ranged in size from 60 million to 100 million base pairs, comprising 40,000 to 80,000 contigs (Supp. Table S1). By both measures, the *T. ambiguus* transcriptome is the smallest, whereas the *T. curvispinosus* transcriptome is the largest. Additional factors like GC content and average contig length are broadly similar across all transcriptomes, indicating consistency within assembly method. Gene content of transcriptomes is somewhat variable, ranging from 18,396 annotated genes in the *T. curvispinosus* transcriptome to 10,206 genes in the *T. pilagens* transcriptome.

*Supplementary Discussion*

**Additional Genes of Interest** Unsurprisingly, given their widespread importance in physiological and behavioral processes in social insects ^79, 80, 81^, several *Vitellogenin*-family genes were found here to be differentially expressed between species and lifestyle. This finding in itself is not novel. Indeed, *Temnothorax* species possess four copies of *Vitellogenin*-family genes ^82^, each with a different caste-specific regulation ^83^. Unusually, however, we find *Vitellogenin-6* (*Vg6*), to be over-expressed within *T. longispinosus* during raid defense and *T. pilagens* and *T. duloticus* during raids. A related study suggests that *Vg6* may be involved in brood cue response thresholds, brood care behavior, and task differentiation in *T. longispinosus* workers. Thus, that *Vg6* is found to be highly up-regulated *during nest defense in a host*, and *during active raiding behavior in slavemakers* is very surprising, given that normal care behavior ceases in hosts under raiding attack, and slavemaker workers have lost the ability to care for brood outright. However, while intriuging, whether or not *Vg6* has been re-purposed within these two slavemaking species in order to modify brood thresholds towards *host* brood is a question best answered by a more gene-targeted approach.

*Vitellogenin-3* (*Vg3*) and *Vitellogenin Receptor* (*VgRec*) were also found here to be differentially expressed. *Vg3* was found to be over-expressed within *T. duloticus* workers out of raiding season and *T. ambiguus* workers during raid defense. In this study, and paralleling the pattern of *Vg3*, *VgRec* was also found here to be up-regulated in *T. ambiguus* during nest defense; as well as in *T. pilagens* and *T. duloticus* in the non-raiding state. The expression patterns observed here, combined with insight previously provided into the functions of *Vg3* and *VgRec* supports previous findings into the raiding propensity of different slavemaker workers. Our findings reinforce the observation that only non-fertile slavemakers participate in raiding behavior, and suggest that *Vg3* has maintained its fertility-related function within *Temnothorax* raiders. Within host *T. ambiguus*, we might conclude from the expression of *Vg3* and *VgRec* that fertile host workers participate more when defending nests against slavemaker aggression; a not-unlikely scenario given that brood nurses – typically located within their own colonies near developing brood - within non-slavemaking *Temnothorax* species are generally more fertile than foragers ^84^.

Surprisingly, several genes relating to mitochondrial oxidative phosphorylation (cytochrome- and NADH dehydrogenase-family) were also found here to be down-regulated within the slavemaker raiding and host defensive behavior. This was unexpected, as we initially predicted that the behavioral states characterized by activity and aggression would also display the up-regulation of genes involved in energy metabolism. This expectation corresponds with a number of earlier studies showing that, across a wide range of taxa, elevated levels of aggression positively correspond with changes in expression of general metabolic genes ^85, 86, 87, 88, 89^. Conversely, however, a number of studies examining social insect workers specifically find the reverse, suggesting that this positive correlation between metabolic gene expression levels and aggression is inverted in energy metabolic genes, with aggressive states being characterized by the down-regulation of these genes ^85^ within the insect worker brain. Our findings certainly align more with these latter observations.

Two additional genes, involved in the response to stress, were also found to be differentially-expressed within slavemaking and/or host species. *Kelch*, found here to be up-regulated in *T. longispinosus* during the non-raiding state and *T. pilagens* during raiding behavior, has previously been described in *Drosophila melanogaster* as being involved in response to oxidative stress ^90^. Oxidative stress responses can be seen as body maintenance or repair and it is likely that hosts shut down such general repair genes when under attack to concentrate on defending their nest. Slavemakers on the other hand, might up-regulate repair mechanisms during raids as these constitute a very active state and they might prepare for metabolic damages by activating genes involved in response to oxidative stress. The second stress-response gene, *MYG1*, has previously been found to be involved in stress response and general activity within *Mus musculus* ^91^. Here, it is up-regulated in *T. curvispinosus* and *T. americanus* outside of defense and raiding behavior, respectively. Together, the expression patterns of these two genes does seem to suggest that, within both slavemaker and host species, the non-raiding state is spent performing bodily maintenance and repair tasks.

Perhaps unsurprisingly, given the antagonistic nature of the behaviors examined here, genes involved in pain response and nociception did show differential expression between behavioral states. Chief among those over-expressed is the cation channel protein *painless*. Within *D. melanogaster*, *painless* is found to be responsible for nociception and response to heat, where *D. melanogaster* with nonfunctional *painless* genes display an impaired pain response ^92^. Here, we find this gene over-expressed within *T. curvispinosus* during nest defense and *T. americanus* during raiding behavior. This is especially intriguing, given that both of these species up-regulate this gene during times of conflict. This observation raises additional questions: under circumstances where injury is likely, is it beneficial to up-regulate the expression of genes responsible for reporting pain? Does this up-regulation result in an individual more sensitive to pain, and thus quicker to respond to painful situations? Results of previous studies, combined with the gene expression data produced here, certainly seem to suggest this. One final note on nociception: while previous studies have shown that *Hymenoptaecin* is strongly over-expressed in injured individuals ^93^, our results indicate that this gene is only up-regulated during the raiding behavior of *T. duloticus,* while showing significant down-regulation during the raiding state of *T. pilagens* and *T. americanus*, and in the host *T. ambiguus*. Thus, while *Hymenoptaecin* is a definitive response to injury in some ant species, its function within *Temnothorax* is much less clear.

*Supplementary References*

79] Raikhel, A. S., Dhadialla, T. S. Accumulation of yolk proteins in insect oocytes. *Annu Rev Entomol*. **37,** 217-251 (1992).

80] Ihle, K. E., Page, R. E., Frederick, K., Fondrk, M. K., Amdam, G.V. Genotype effect on regulation of behavior by vitellogenin supports reproductive origin of honeybee foraging bias. *Anim Behav*. **79,** 1001-1006 (2010).

81] Samela, H., Sundström, L. Vitellogenin in inflammation and immunity in social insects. *Inflammation and Cell Signaling*. 4: e1506. Doi:10.14800/ics.1506 (2017).

82] Wurm, Y., *et al*. The genome of the fire ant *Solenopsis invicta*. *Proc Natl Acad Sci USA*. **108,** 5679–5684 (2011).

83] Corona, M., Libbrecht, R., Wurm, Y., Riba-Grognuz, O., Studer, R. A., Keller, L. Vitellogenin underwent subfunctionalization to acquire caste and behavioral specific expression in the harvester ant *Pogonomyrmex barbatus*. *PLoS Genet*. **9** (2013).

84] Konrad, M., Pamminger, T., Foitzik, S. Two pathways ensuring social harmony. ***Naturwissenschaften*.** **99,** 627–636 (2012).

85] Alaux, C., *et al*. Regulation of brain gene expression in honey bees by brood pheromone. *Genes Brain Behav*. **8,** 309-319 (2009).

86] Ayroles, J. F., *et al*. Systems of complex genetics in *Drosophila melanogaster*. *Nat. Genet*. **41,** 299–307 (2009).

87] Edwards, A., Zwarts, L., Yamamoto, A., Callaerts, P., Mackay, T. Mutations in many genes affect aggressive behavior in *Drosophila melanogaster*. *BMC Biol*. **7,** 29 (2009).

88] Li-Byarlay, H., Rittschof, C. C., Massey, J. H., Pittendrigh, B. R., Robinson, G. E. Socially responsive effects of brain oxidative metabolism on aggression. *Proc Natl Acad Sci USA*. **111,** 12533–12537 (2014).

89] Helmkampf, M., Mikheyev, A. S., Kang, Y., Fewell, J., Gadau, J. Gene expression and variation in social aggression by queens of the harvester ant *Pogonomyrmex californicus*. *Mol Ecol*. **25,** 3716–3730 (2016).

90] Sekine, Y., *et al*. The Kelch Repeat Protein KLHDC10 Regulates Oxidative Stress-Induced ASK1 Activation by Suppressing PP5. *Mol Cell*. **48,** 692–704 (2012).

91] Philips, M. A. Myg1-deficient mice display alterations in stress-induced responses and reduction of sex-dependent behavioural differences. *Behav Brain Res*. **207,** 182–195 (2012).

92] Tracey, W. D., Wilson, R. I., Laurent, G., Benzer, S. painless, a *Drosophila* gene essential for nociception. *Cell*. **113,** 261–273 (2003).

93] Von Wyschetzki, K., Lowack, H., Heinze, J. Transcriptomic response to injury sheds light on the physiological costs of reproduction in ant queens. *Mol Ecol*. **25,** 1972-1985 (2016).

*Supplementary Tables and Figures*

**Supplementary Table S1.** Statistics of the final transcriptomes used within this analysis. Information displayed here obtained from transcriptomes produced through meta-assembly using CLC Workbench and MIRA, refined with CD-Hit and Qualimap.

|  | ***T. ambiguus*** | ***T. curvispinosus*** | ***T. longispinosus*** | ***T. americanus*** | ***T. duloticus*** | ***T. pilagens*** |
| --- | --- | --- | --- | --- | --- | --- |
| **Assembly Statistics** |  |  |  |  |  |  |
| Total length of sequence | 63,020,600 | 96,205,599 | 75,609,790 | 73,000,788 | 77,978,773 | 65,405,297 |
| Total number of contigs | 43,664 | 79,277 | 56,122 | 54,054 | 57,994 | 48,783 |
| N25 stats | 6,115 | 5,926 | 6,506 | 6,511 | 6,795 | 6,442 |
| N50 stats | 3,385 | 2,973 | 3,521 | 3,531 | 3,606 | 3,504 |
| N75 stats | 1,410 | 1,017 | 1,302 | 1,306 | 1,253 | 1,298 |
| Total GC count | 26,220,765 | 40,730,944 | 31,501,177 | 30,697,888 | 32,286,868 | 26,968,818 |
| GC % | 41.61 % | 42.34 % | 41.66 % | 42.05 % | 41.40 % | 41.23 % |
| Average Contig Length | 1,443 | 1,214 | 1,347 | 1,351 | 1,345 | 1,341 |
|  |  |  |  |  |  |  |
| **Blast and Filtration Results** |  |  |  |  |  |  |
| Initial number of gene annotations | 18,501 | 31,636 | 21,626 | 18,914 | 17,765 | 16,433 |
| Final number of single gene annotations | 11,350 | 18,396 | 12,240 | 11,809 | 10,407 | 10,206 |
| % of genes filtered | 38.65% | 41.85% | 43.40% | 37.56% | 41.42% | 37.89% |

**Supplementary Table S2.** Extensive comparison of all assembly methods tested during the course of this project (additional file).

**Supplementary Table S3.** Only significantly differentially-expressed contigs for all species (additional file).

**Supplementary Table S4.** Six-tabbed table containing complete expression data for all species and all contigs (additional file).

**Supplementary Table S5.** Enriched functions of contigs within WGCNA module Slavemaker Module 1. Green rows indicate significant enrichment.

| GO.ID | Term | Annotated | Significant | Expected | Fisher |
| --- | --- | --- | --- | --- | --- |
| GO:0006412 | translation | 84 | 6 | 1.44 | 0.0026 |
| GO:0071918 | urea transmembrane transport | 1 | 1 | 0.02 | 0.0172 |
| GO:0006783 | heme biosynthetic process | 1 | 1 | 0.02 | 0.0172 |
| GO:0006685 | sphingomyelin catabolic process | 1 | 1 | 0.02 | 0.0172 |
| GO:0019441 | tryptophan catabolic process to kynureni... | 1 | 1 | 0.02 | 0.0172 |
| GO:0006750 | glutathione biosynthetic process | 2 | 1 | 0.03 | 0.0341 |
| GO:0006629 | lipid metabolic process | 65 | 4 | 1.12 | 0.0463 |
| GO:0042981 | regulation of apoptotic process | 3 | 1 | 0.05 | 0.0507 |
| GO:0015986 | ATP synthesis coupled proton transport | 4 | 1 | 0.07 | 0.067 |
| GO:0015936 | coenzyme A metabolic process | 4 | 1 | 0.07 | 0.067 |
| GO:0006334 | nucleosome assembly | 6 | 1 | 0.1 | 0.0989 |
| GO:0008299 | isoprenoid biosynthetic process | 7 | 1 | 0.12 | 0.1144 |
| GO:0006099 | tricarboxylic acid cycle | 10 | 1 | 0.17 | 0.1594 |
| GO:0008152 | metabolic process | 1483 | 31 | 25.48 | 0.1815 |
| GO:0043401 | steroid hormone mediated signaling pathw... | 16 | 1 | 0.27 | 0.2429 |
| GO:0006313 | transposition, DNA-mediated | 17 | 1 | 0.29 | 0.256 |
| GO:0006030 | chitin metabolic process | 19 | 1 | 0.33 | 0.2815 |
| GO:0055114 | oxidation-reduction process | 177 | 5 | 3.04 | 0.2823 |
| GO:0016579 | protein deubiquitination | 20 | 1 | 0.34 | 0.294 |
| GO:0035023 | regulation of Rho protein signal transdu... | 21 | 1 | 0.36 | 0.3062 |
| GO:0006470 | protein dephosphorylation | 22 | 1 | 0.38 | 0.3182 |
| GO:0006355 | regulation of transcription, DNA-templat... | 209 | 4 | 3.59 | 0.4898 |
| GO:0016192 | vesicle-mediated transport | 47 | 1 | 0.81 | 0.5609 |
| GO:0006810 | transport | 370 | 6 | 6.36 | 0.6108 |
| GO:0055085 | transmembrane transport | 166 | 4 | 2.85 | 0.6758 |
| GO:0006468 | protein phosphorylation | 161 | 2 | 2.77 | 0.7769 |
| GO:0006396 | RNA processing | 95 | 1 | 1.63 | 0.8139 |
| GO:0071103 | DNA conformation change | 13 | 1 | 0.22 | 1 |
| GO:0019438 | aromatic compound biosynthetic process | 290 | 6 | 4.98 | 1 |
| GO:0015672 | monovalent inorganic cation transport | 37 | 1 | 0.64 | 1 |
| GO:0019439 | aromatic compound catabolic process | 23 | 1 | 0.4 | 1 |
| GO:0032196 | transposition | 17 | 1 | 0.29 | 1 |
| GO:0008219 | cell death | 8 | 1 | 0.14 | 1 |
| GO:0019219 | regulation of nucleobase-containing comp... | 213 | 4 | 3.66 | 1 |
| GO:0050794 | regulation of cellular process | 540 | 6 | 9.28 | 1 |
| GO:0043412 | macromolecule modification | 318 | 4 | 5.46 | 1 |
| GO:0044260 | cellular macromolecule metabolic process | 856 | 16 | 14.71 | 1 |
| GO:0015840 | urea transport | 1 | 1 | 0.02 | 1 |
| GO:0006022 | aminoglycan metabolic process | 25 | 1 | 0.43 | 1 |

**Supplementary Table S6.** Enriched functions of contigs within WGCNA module Slavemaker Module 7. Green rows indicate significant enrichment.

| GO.ID | Term | Annotated | Significant | Expected | Fisher |
| --- | --- | --- | --- | --- | --- |
| GO:0006412 | translation | 84 | 8 | 1.52 | 0.00029 |
| GO:0006979 | response to oxidative stress | 8 | 2 | 0.14 | 0.00832 |
| GO:0000462 | maturation of SSU-rRNA from tricistronic... | 1 | 1 | 0.02 | 0.01806 |
| GO:0035434 | copper ion transmembrane transport | 1 | 1 | 0.02 | 0.01806 |
| GO:0006430 | lysyl-tRNA aminoacylation | 1 | 1 | 0.02 | 0.01806 |
| GO:0006108 | malate metabolic process | 1 | 1 | 0.02 | 0.01806 |
| GO:0001678 | cellular glucose homeostasis | 2 | 1 | 0.04 | 0.0358 |
| GO:0046835 | carbohydrate phosphorylation | 2 | 1 | 0.04 | 0.0358 |
| GO:0006850 | mitochondrial pyruvate transport | 2 | 1 | 0.04 | 0.0358 |
| GO:0010508 | positive regulation of autophagy | 2 | 1 | 0.04 | 0.0358 |
| GO:0008272 | sulfate transport | 2 | 1 | 0.04 | 0.0358 |
| GO:0006744 | ubiquinone biosynthetic process | 3 | 1 | 0.05 | 0.05324 |
| GO:0016226 | iron-sulfur cluster assembly | 4 | 1 | 0.07 | 0.07036 |
| GO:0035176 | social behavior | 4 | 1 | 0.07 | 0.07036 |
| GO:0006355 | regulation of transcription, DNA-templat... | 209 | 7 | 3.77 | 0.07673 |
| GO:0006414 | translational elongation | 5 | 1 | 0.09 | 0.08718 |
| GO:0030203 | glycosaminoglycan metabolic process | 6 | 1 | 0.11 | 0.1037 |
| GO:0006334 | nucleosome assembly | 6 | 1 | 0.11 | 0.1037 |
| GO:0006298 | mismatch repair | 9 | 1 | 0.16 | 0.15154 |
| GO:0006096 | glycolytic process | 9 | 1 | 0.16 | 0.15154 |
| GO:0006099 | tricarboxylic acid cycle | 10 | 1 | 0.18 | 0.16693 |
| GO:0009058 | biosynthetic process | 516 | 18 | 9.32 | 0.19698 |
| GO:0008152 | metabolic process | 1483 | 32 | 26.79 | 0.21116 |
| GO:0016579 | protein deubiquitination | 20 | 1 | 0.36 | 0.30656 |
| GO:0006511 | ubiquitin-dependent protein catabolic pr... | 35 | 1 | 0.63 | 0.47419 |
| GO:0007186 | G-protein coupled receptor signaling pat... | 89 | 2 | 1.61 | 0.4831 |
| GO:0005975 | carbohydrate metabolic process | 68 | 2 | 1.23 | 0.48955 |
| GO:0055114 | oxidation-reduction process | 177 | 4 | 3.2 | 0.54918 |
| GO:0006508 | proteolysis | 157 | 3 | 2.84 | 0.62106 |
| GO:0055085 | transmembrane transport | 166 | 2 | 3 | 0.92439 |
| GO:0007165 | signal transduction | 295 | 3 | 5.33 | 0.97986 |
| GO:0071103 | DNA conformation change | 13 | 1 | 0.23 | 1 |
| GO:0019438 | aromatic compound biosynthetic process | 290 | 7 | 5.24 | 1 |
| GO:0019725 | cellular homeostasis | 26 | 1 | 0.47 | 1 |
| GO:0006364 | rRNA processing | 13 | 1 | 0.23 | 1 |
| GO:0019219 | regulation of nucleobase-containing comp... | 213 | 7 | 3.85 | 1 |
| GO:0050794 | regulation of cellular process | 540 | 11 | 9.75 | 1 |
| GO:0043412 | macromolecule modification | 318 | 1 | 5.74 | 1 |
| GO:0044260 | cellular macromolecule metabolic process | 856 | 18 | 15.46 | 1 |
| GO:0006022 | aminoglycan metabolic process | 25 | 1 | 0.45 | 1 |
| GO:0044262 | cellular carbohydrate metabolic process | 13 | 1 | 0.23 | 1 |

**Supplementary Table S7.** Enriched functions of contigs within WGCNA module Host Module 9. Green rows indicate significant enrichment.

| GO.ID | Term | Annotated | Significant | Expected | Fisher |
| --- | --- | --- | --- | --- | --- |
| GO:0006629 | lipid metabolic process | 70 | 6 | 1.91 | 0.017 |
| GO:0008299 | isoprenoid biosynthetic process | 9 | 2 | 0.25 | 0.023 |
| GO:0045116 | protein neddylation | 1 | 1 | 0.03 | 0.027 |
| GO:0007517 | muscle organ development | 1 | 1 | 0.03 | 0.027 |
| GO:0019510 | S-adenosylhomocysteine catabolic process | 1 | 1 | 0.03 | 0.027 |
| GO:0042073 | intraciliary transport | 1 | 1 | 0.03 | 0.027 |
| GO:0006685 | sphingomyelin catabolic process | 1 | 1 | 0.03 | 0.027 |
| GO:1902751 | positive regulation of cell cycle G2/M p... | 1 | 1 | 0.03 | 0.027 |
| GO:0045823 | positive regulation of heart contraction | 1 | 1 | 0.03 | 0.027 |
| GO:0006529 | asparagine biosynthetic process | 1 | 1 | 0.03 | 0.027 |
| GO:0019441 | tryptophan catabolic process to kynureni... | 1 | 1 | 0.03 | 0.027 |
| GO:0019509 | L-methionine salvage from methylthioaden... | 2 | 1 | 0.05 | 0.054 |
| GO:0000256 | allantoin catabolic process | 2 | 1 | 0.05 | 0.054 |
| GO:0007205 | protein kinase C-activating G-protein co... | 3 | 1 | 0.08 | 0.08 |
| GO:0006730 | one-carbon metabolic process | 4 | 1 | 0.11 | 0.105 |
| GO:0006487 | protein N-linked glycosylation | 4 | 1 | 0.11 | 0.105 |
| GO:0006098 | pentose-phosphate shunt | 4 | 1 | 0.11 | 0.105 |
| GO:0051301 | cell division | 4 | 1 | 0.11 | 0.105 |
| GO:0006298 | mismatch repair | 4 | 1 | 0.11 | 0.105 |
| GO:0007608 | sensory perception of smell | 26 | 2 | 0.71 | 0.157 |
| GO:0000956 | nuclear-transcribed mRNA catabolic proce... | 7 | 1 | 0.19 | 0.176 |
| GO:0007156 | homophilic cell adhesion via plasma memb... | 7 | 1 | 0.19 | 0.176 |
| GO:0006302 | double-strand break repair | 7 | 1 | 0.19 | 0.176 |
| GO:0051726 | regulation of cell cycle | 12 | 2 | 0.33 | 0.196 |
| GO:0006270 | DNA replication initiation | 8 | 1 | 0.22 | 0.199 |
| GO:0007169 | transmembrane receptor protein tyrosine ... | 8 | 1 | 0.22 | 0.199 |
| GO:0009190 | cyclic nucleotide biosynthetic process | 9 | 1 | 0.25 | 0.221 |
| GO:0006139 | nucleobase-containing compound metabolic... | 588 | 18 | 16.05 | 0.257 |
| GO:0007155 | cell adhesion | 20 | 2 | 0.55 | 0.3 |
| GO:0009058 | biosynthetic process | 546 | 16 | 14.9 | 0.32 |
| GO:0006355 | regulation of transcription, DNA-templat... | 204 | 7 | 5.57 | 0.32 |
| GO:0043401 | steroid hormone mediated signaling pathw... | 14 | 1 | 0.38 | 0.322 |
| GO:0007018 | microtubule-based movement | 18 | 2 | 0.49 | 0.372 |
| GO:0006869 | lipid transport | 17 | 1 | 0.46 | 0.376 |
| GO:0006352 | DNA-templated transcription, initiation | 18 | 1 | 0.49 | 0.393 |
| GO:0006470 | protein dephosphorylation | 20 | 1 | 0.55 | 0.426 |
| GO:0035556 | intracellular signal transduction | 91 | 3 | 2.48 | 0.456 |
| GO:0007165 | signal transduction | 270 | 7 | 7.37 | 0.525 |
| GO:0008152 | metabolic process | 1535 | 46 | 41.9 | 0.526 |
| GO:0006508 | proteolysis | 175 | 5 | 4.78 | 0.527 |
| GO:0006468 | protein phosphorylation | 141 | 4 | 3.85 | 0.544 |
| GO:0006281 | DNA repair | 48 | 3 | 1.31 | 0.634 |
| GO:0055114 | oxidation-reduction process | 166 | 4 | 4.53 | 0.676 |
| GO:0016192 | vesicle-mediated transport | 55 | 1 | 1.5 | 0.786 |
| GO:0055085 | transmembrane transport | 158 | 3 | 4.31 | 0.818 |
| GO:0005975 | carbohydrate metabolic process | 78 | 1 | 2.13 | 0.889 |
| GO:0006811 | ion transport | 104 | 1 | 2.84 | 0.947 |
| GO:0006396 | RNA processing | 105 | 1 | 2.87 | 0.949 |
| GO:0003008 | system process | 34 | 3 | 0.93 | 1 |
| GO:0072521 | purine-containing compound metabolic pro... | 51 | 1 | 1.39 | 1 |
| GO:0051716 | cellular response to stimulus | 321 | 10 | 8.76 | 1 |
| GO:0046907 | intracellular transport | 73 | 1 | 1.99 | 1 |
| GO:0072523 | purine-containing compound catabolic pro... | 2 | 1 | 0.05 | 1 |
| GO:0010033 | response to organic substance | 23 | 1 | 0.63 | 1 |
| GO:0006810 | transport | 376 | 7 | 10.26 | 1 |
| GO:0072524 | pyridine-containing compound metabolic p... | 19 | 1 | 0.52 | 1 |
| GO:0009889 | regulation of biosynthetic process | 209 | 7 | 5.7 | 1 |
| GO:0006259 | DNA metabolic process | 114 | 4 | 3.11 | 1 |
| GO:0051186 | cofactor metabolic process | 41 | 1 | 1.12 | 1 |
| GO:1902749 | regulation of cell cycle G2/M phase tran... | 1 | 1 | 0.03 | 1 |
| GO:0019438 | aromatic compound biosynthetic process | 294 | 9 | 8.03 | 1 |
| GO:0044106 | cellular amine metabolic process | 3 | 1 | 0.08 | 1 |
| GO:0009755 | hormone-mediated signaling pathway | 14 | 1 | 0.38 | 1 |

**Supplementary Table S8.** Two-tabbed table containing those GO functions significantly over-represented within slavemakers out of raiding season and during raid, which are either specific to one species or are shared by multiple species (additional file).

**
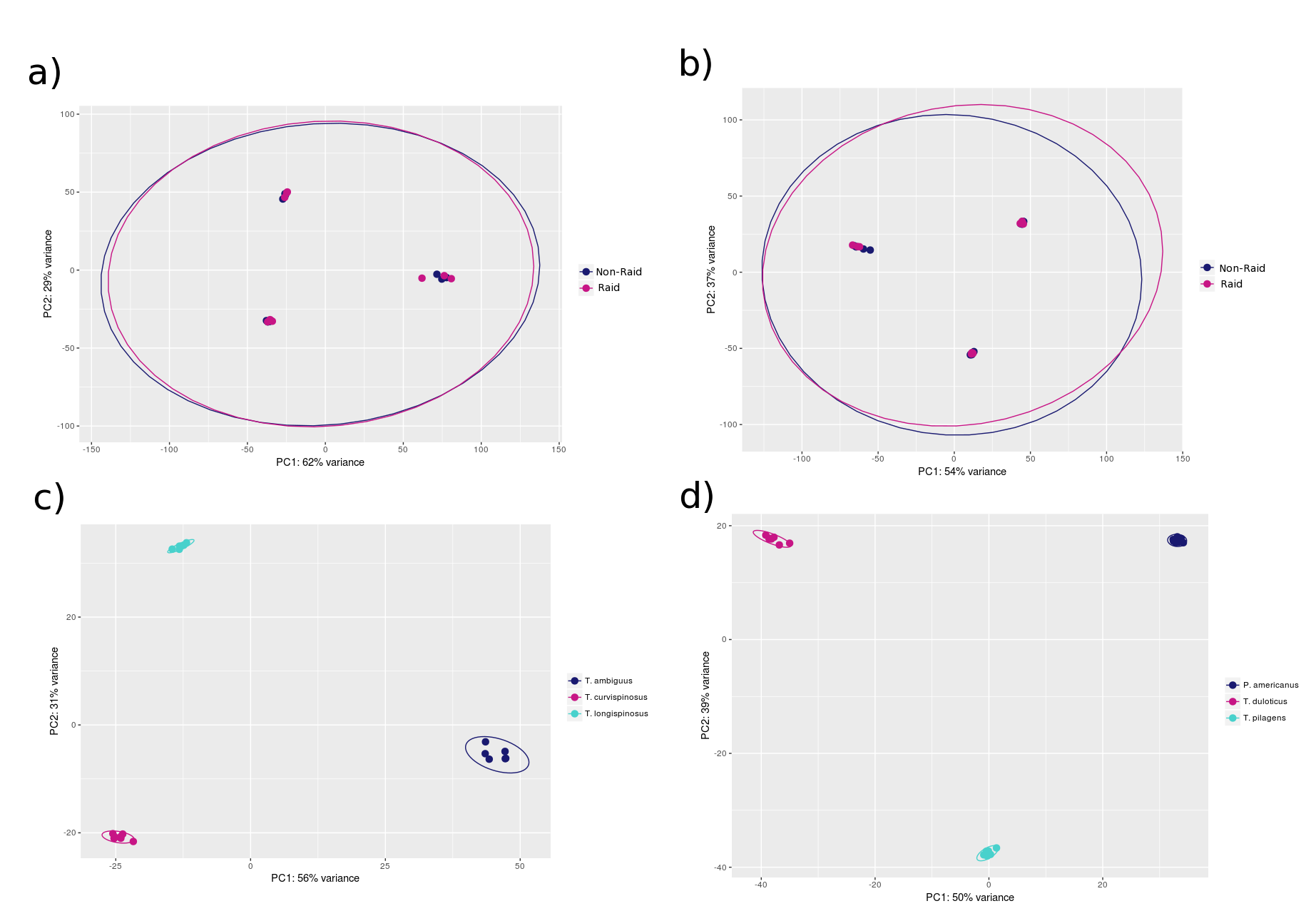
**

**Supplementary Figure S1.** a) PCA plot of normalized read counts of clustered *host* orthologous contigs with a focus on physiological state of over-expression. Samples here do not group by physiological state, but instead by species. b) PCA plot of normalized read counts of clustered *slavemaker* orthologous contigs with a focus on physiological state of over-expression. Samples here do not group by physiological state, but instead by species. c) PCA plot of normalized read counts of clustered *host* orthologous contigs with a focus on species. Grouping is driven primarily by species. d) PCA plot of normalized read counts of clustered *slavemaker* orthologous contigs with a focus on species. Grouping here also appears to be strongly species-driven.


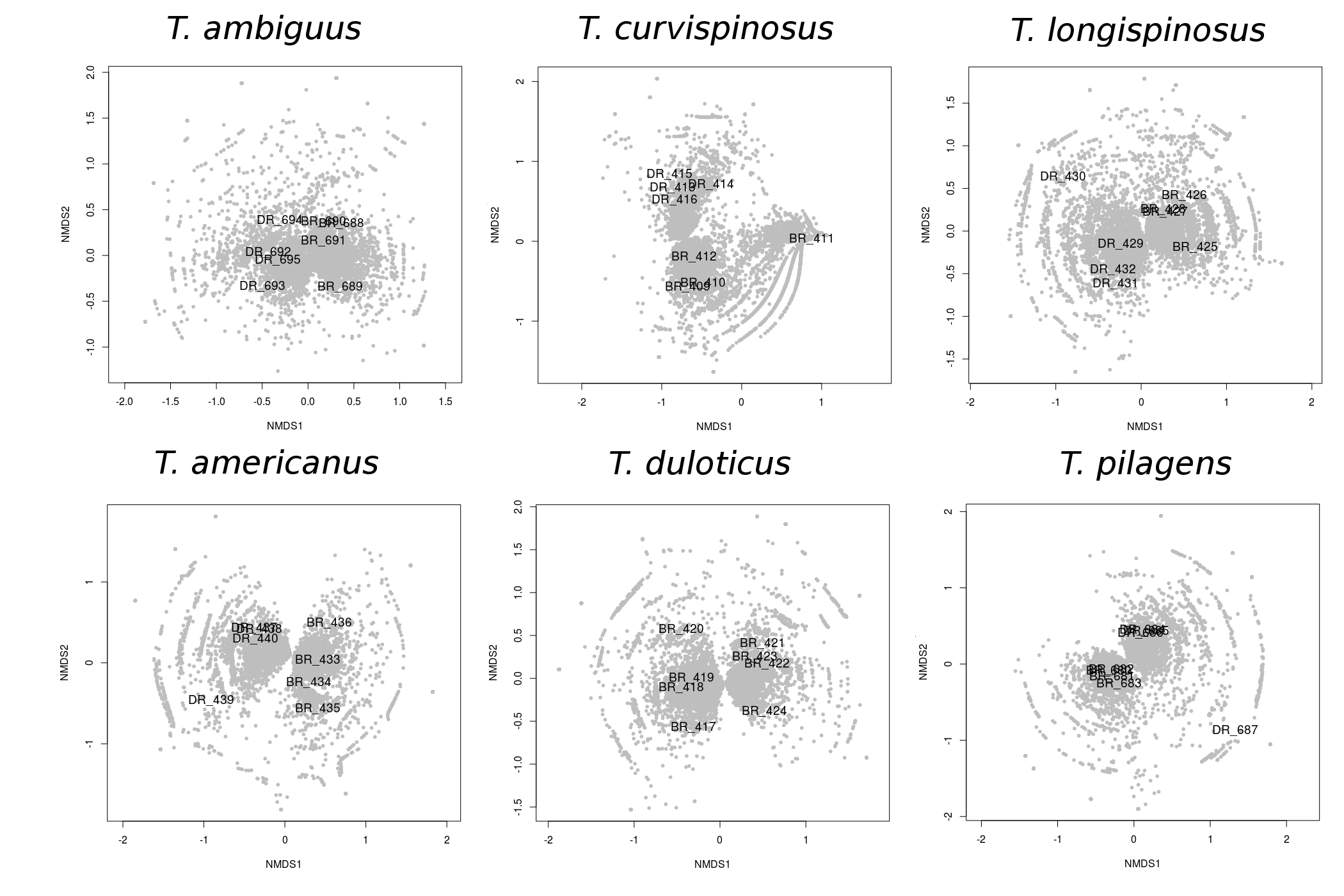


**Supplementary Figure S2.** Initial NMDS analysis including all replicates before removal of outliers. Eight replicates per species, with four out-of-raiding-season samples and four during raid samples per slavemaking species and four before nest defense and four during nest defense samples per host species.

**
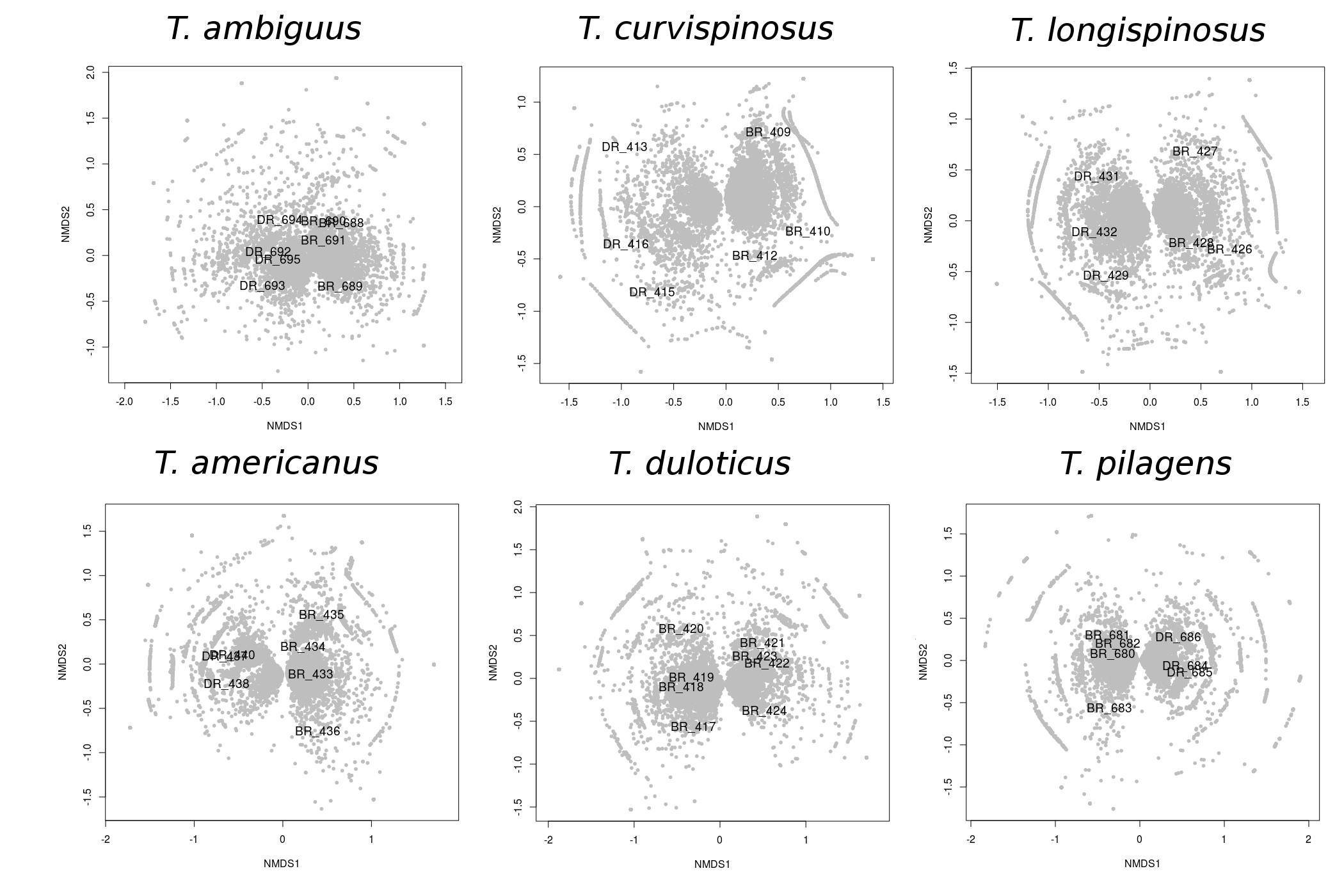
**

**Supplementary Figure S3.** Final NMDS analysis after the removal of six outlying replicates. Outlying replicates: *T. americanus* – DR_439; *T. curvispinosus* – BR_411 and DR_414; *T. longispinosus* – BR_425 and DR_430; *T. pilagens* – DR_687.


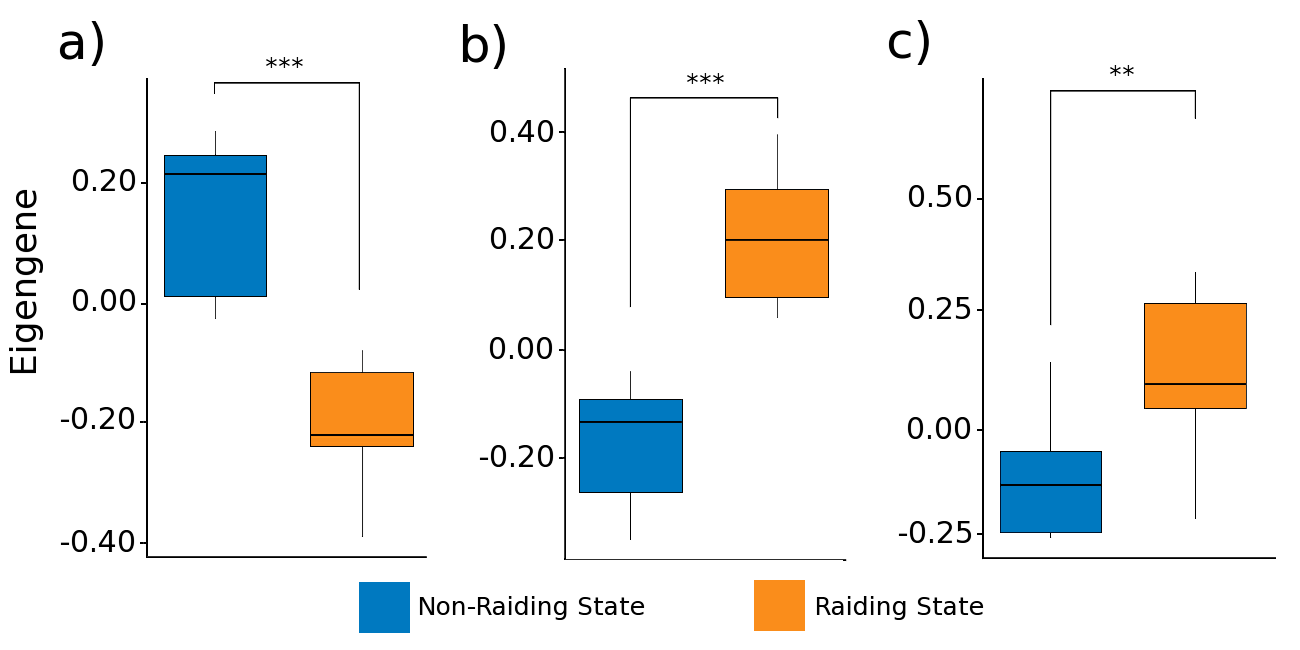


**Supplementary Figure S4.** Kruskal-Wallis tests of WGCNA modules previously determined to be significantly associated with a phenotypic state (fig. 5). a) slavemaker Module 1, p-value < 0.005; b) slavemaker Module 7, p-value < 0.005; and c) host Module 9, p-value = 0.005.


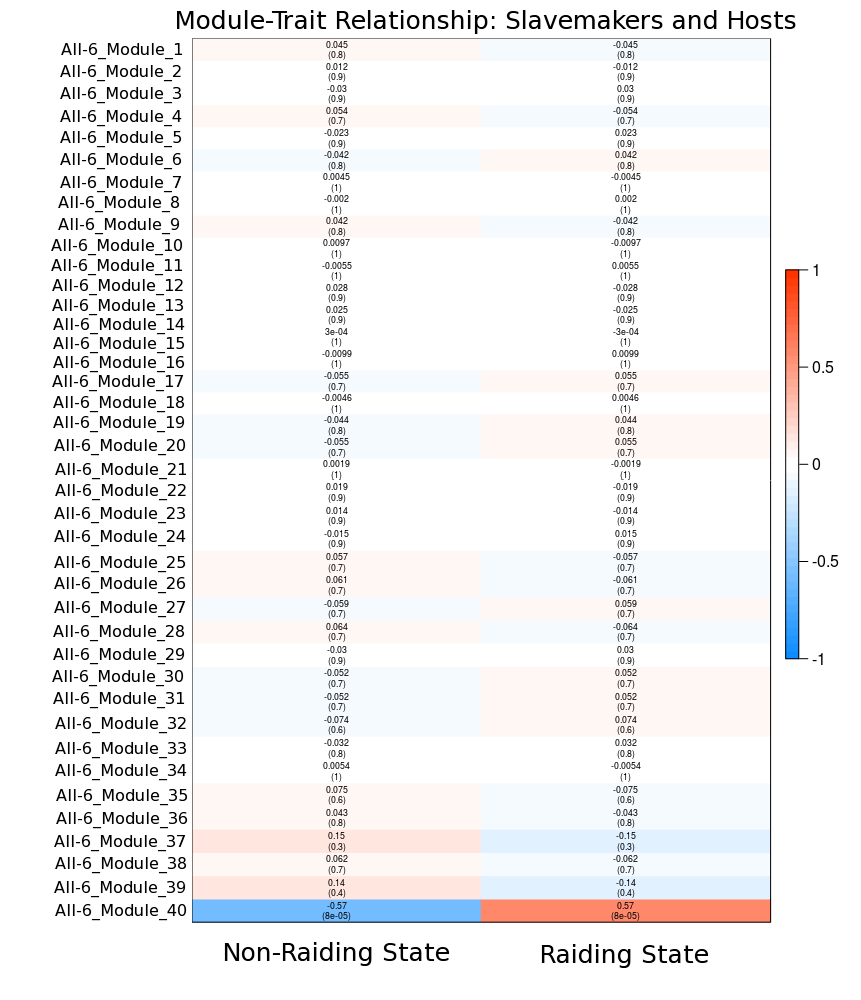


**Supplementary Figure S5.** Module-Trait relationships within all six species herein examined. No contig modules showed significant patterns of shared expression - likely due to the dissimilarity between slavemaker raiding and host defensive behaviors – with the exception of All-6_Module-40, which is an “everything else” group of contigs that did not fit into one of the other modules.

**Functional Enrichment**


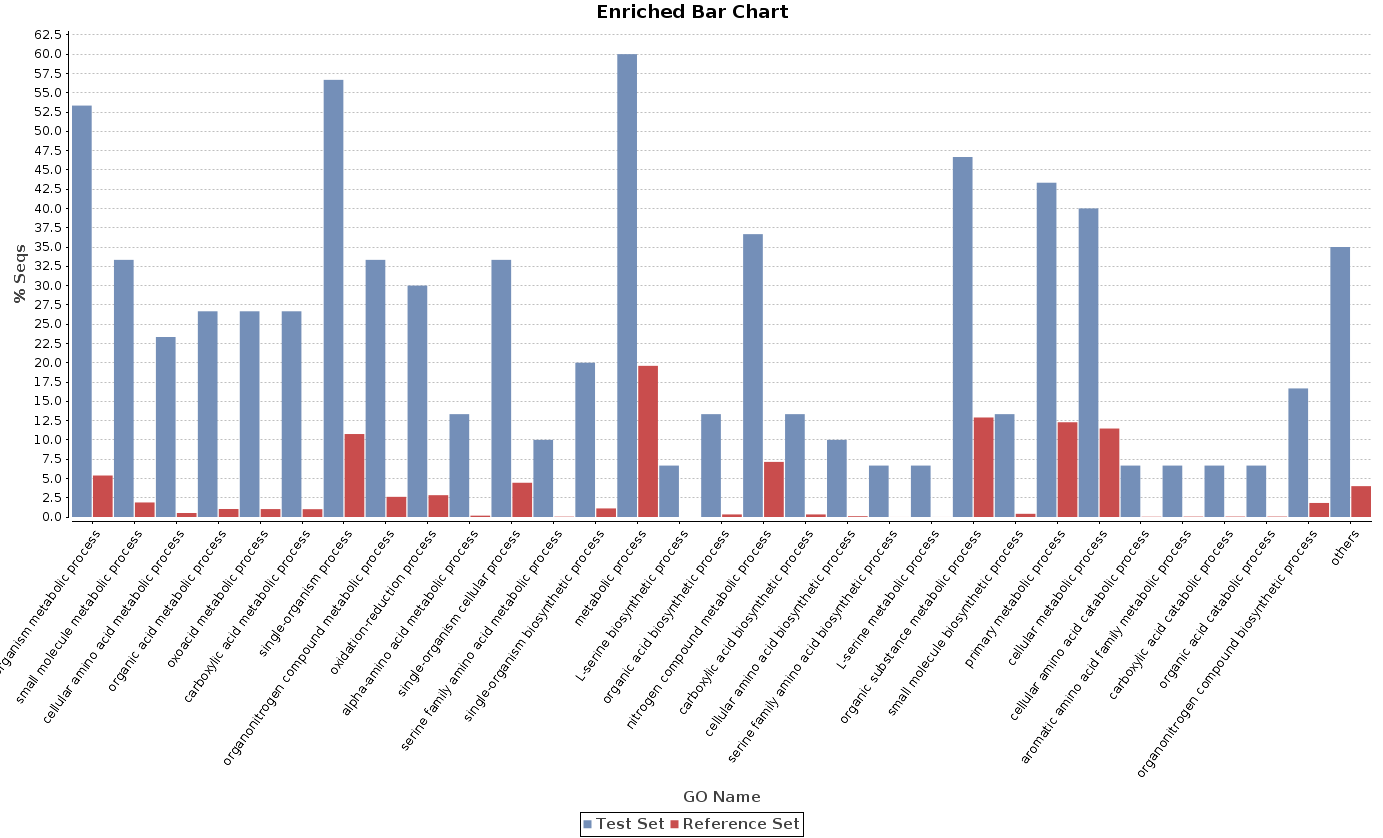


**Supplementary Figure S6.** Functional categories of genes up-regulated *only* by host *T. ambiguus*, before engaging in nest defense behavior (Test Set), compared to the functions of the *T. ambiguus* transcriptome as a whole (Reference Set).


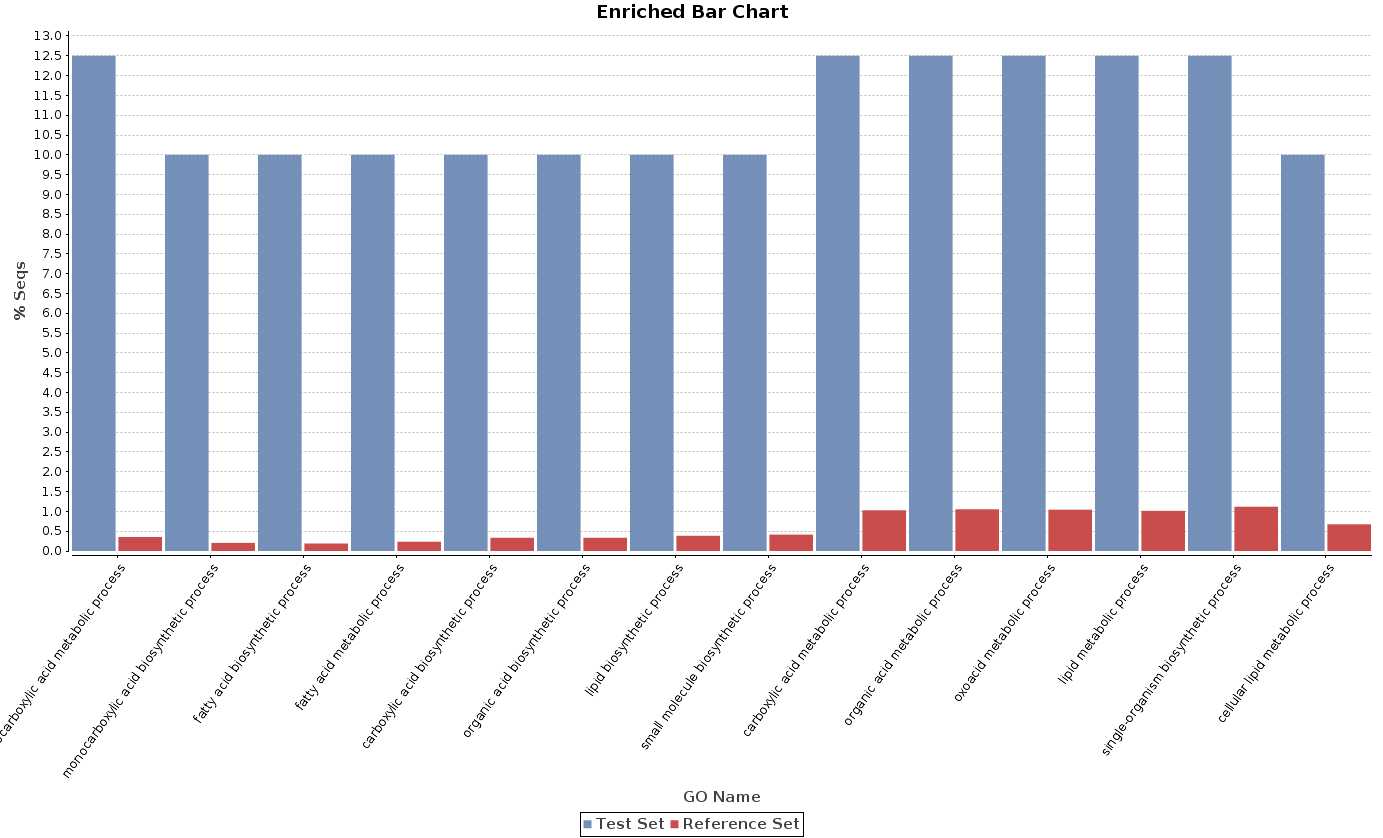


**Supplementary Figure S7.** Functional categories of genes up-regulated *only* by host *T. ambiguus*, during nest defense behavior (Test Set), compared to the functions of the *T. ambiguus* transcriptome as a whole (Reference Set).


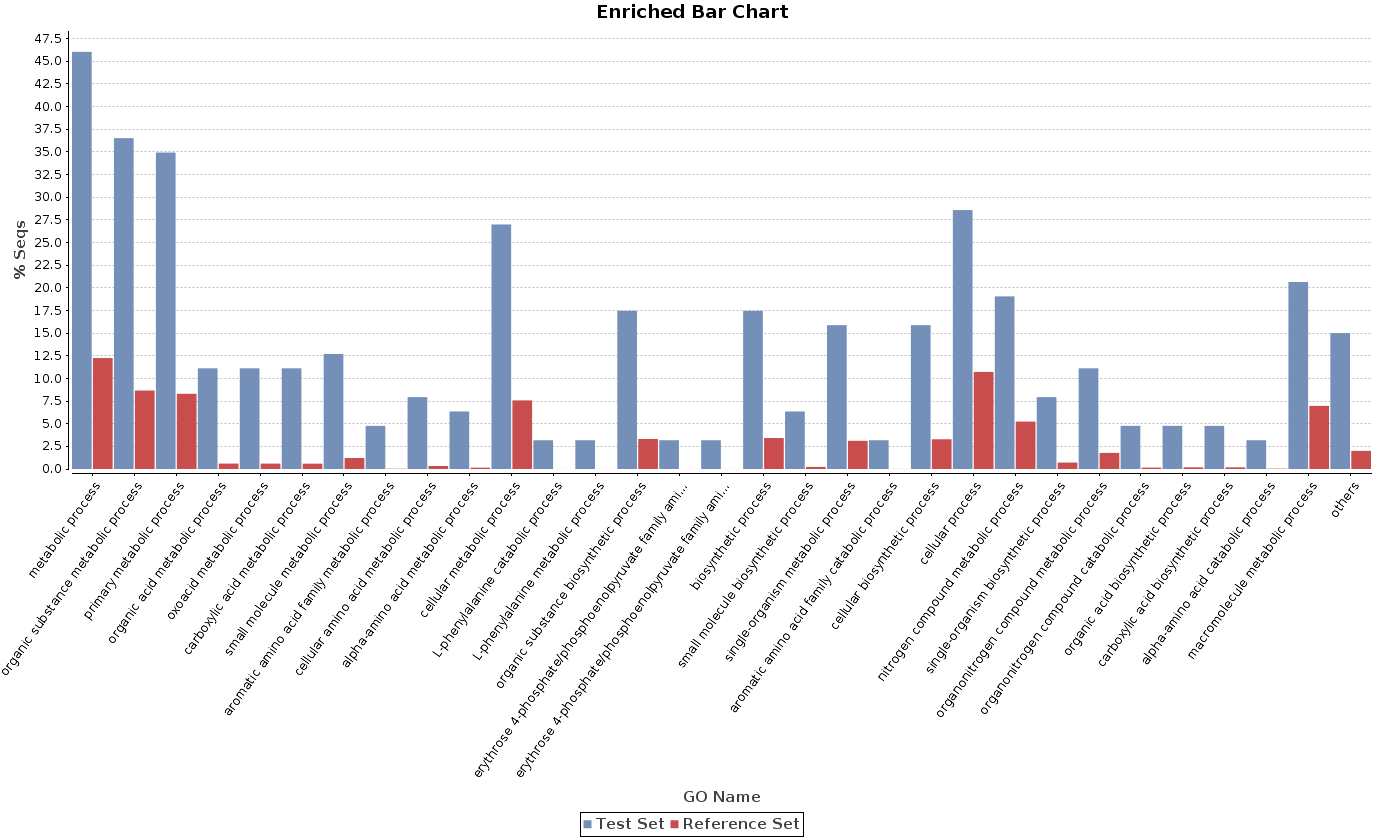


**Supplementary Figure S8.** Functional categories of genes up-regulated *only* by slavemaker *T. americanus*, while out of raiding season (Test Set), compared to the functions of the *T. americanus* transcriptome as a whole (Reference Set).


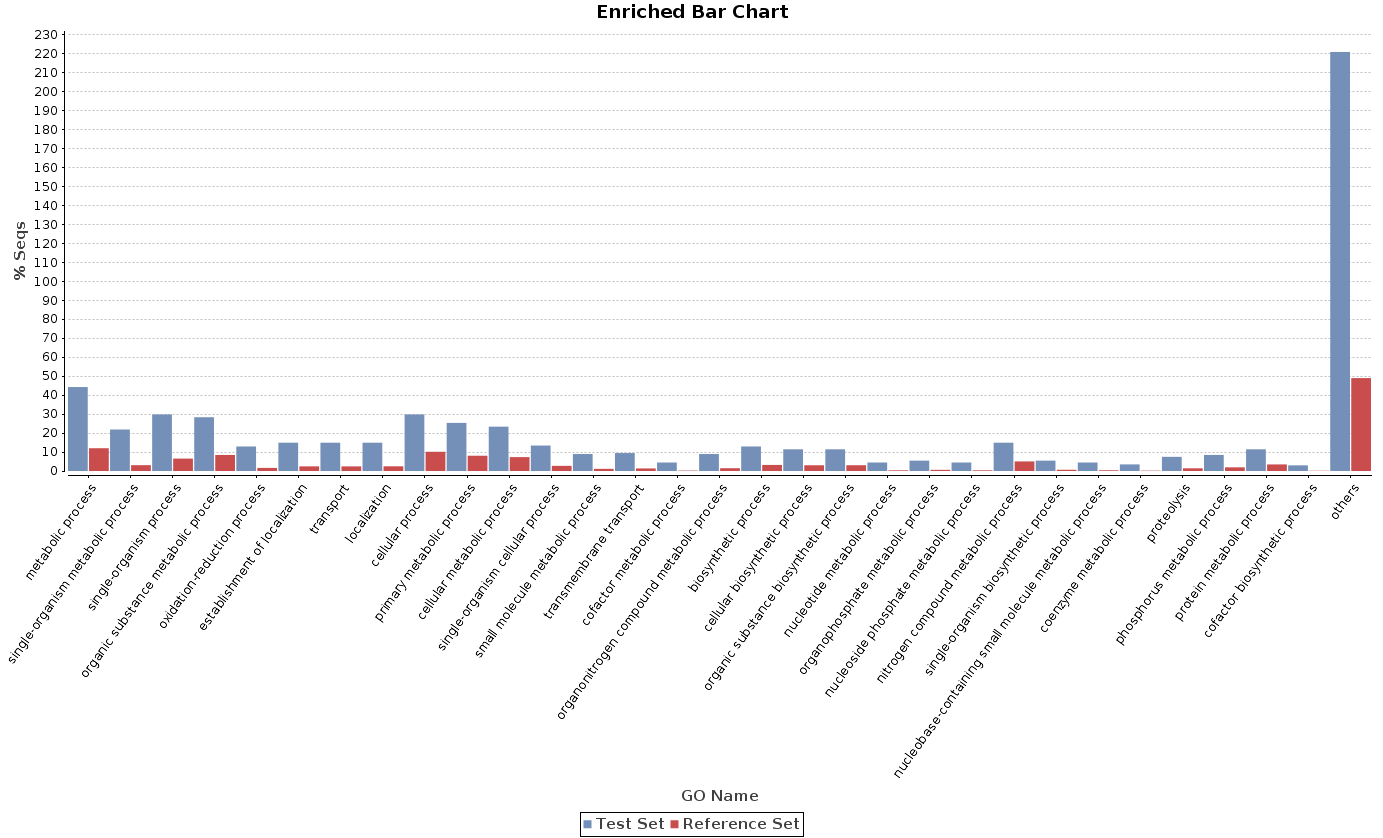


**Supplementary Figure S9.** Functional categories of genes up-regulated *only* by slavemaker *T. pilagens*, while out of raiding season (Test Set), compared to the functions of the *T. pilagens* transcriptome as a whole (Reference Set).


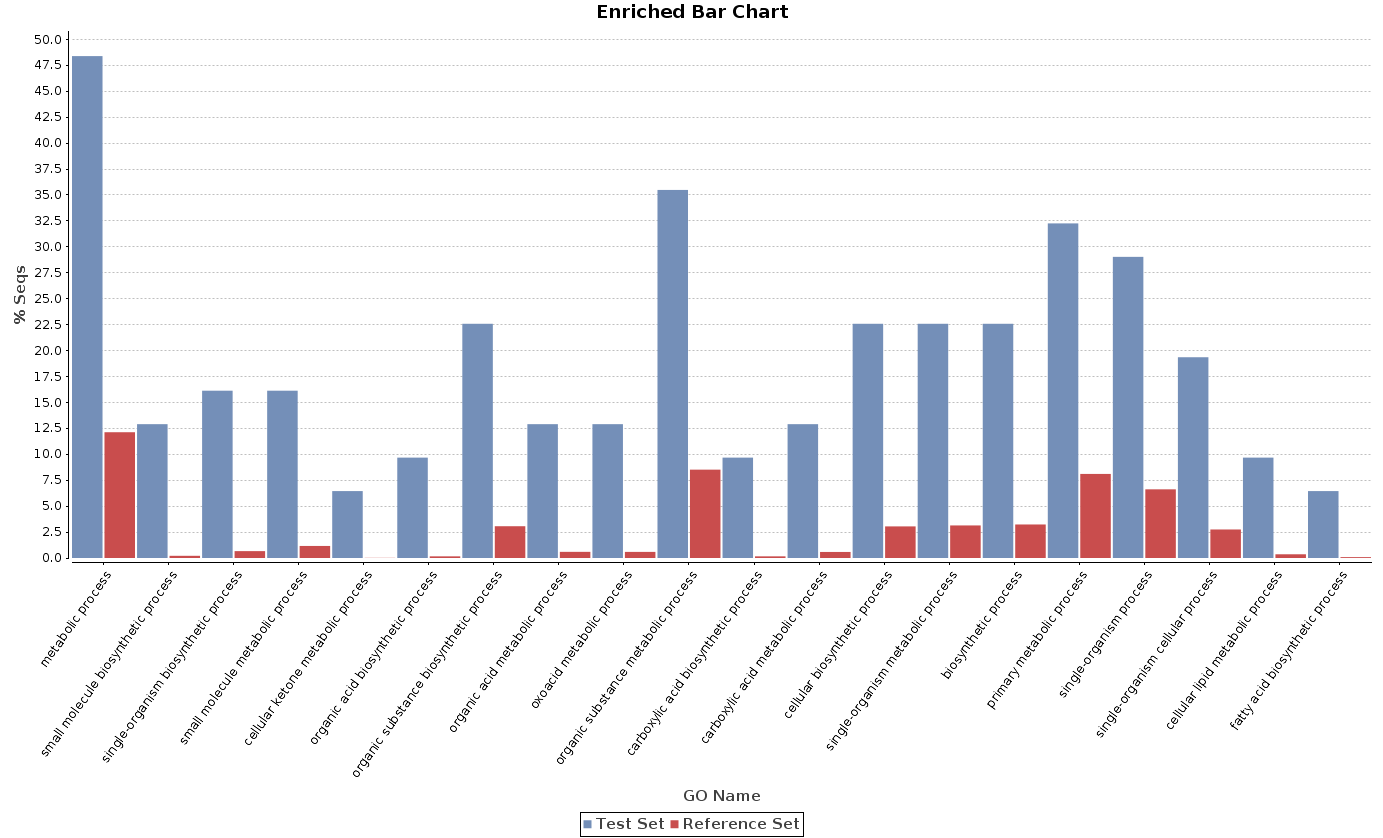


**Supplementary Figure S10.** Functional categories of genes up-regulated by *all* slavemaking species while out of raiding season (Test Set), compared to the functions of the most well annotated slavemaker transcriptome of *T. pilagens*. (Reference Set).


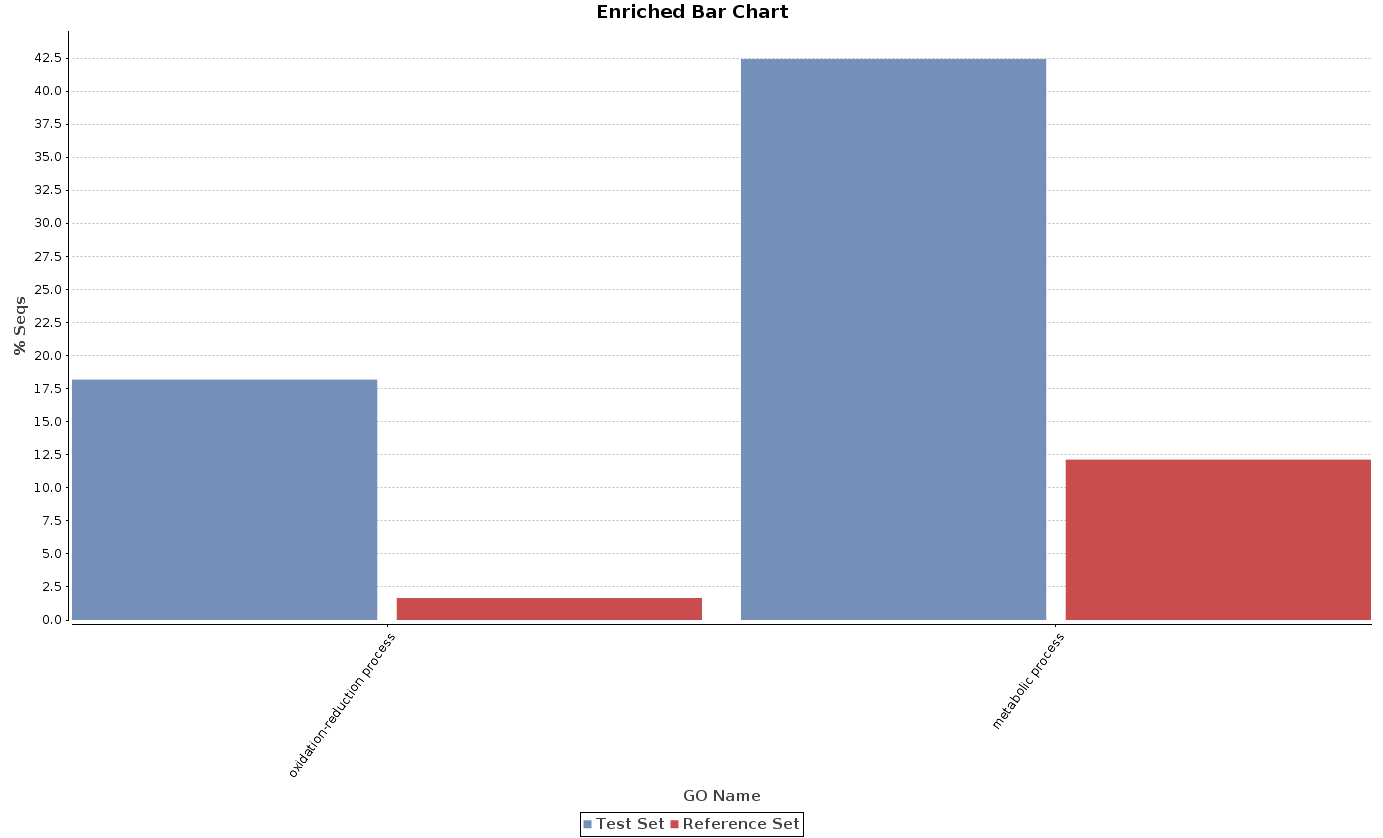


**Supplementary Figure S11.** Functional categories of genes up-regulated by *both* slavemakers *T. pilagens* and *T. duloticus* before engaging in raiding behavior (Test Set), compared to the functions of the *T. pilagens* transcriptome as a whole (Reference Set).


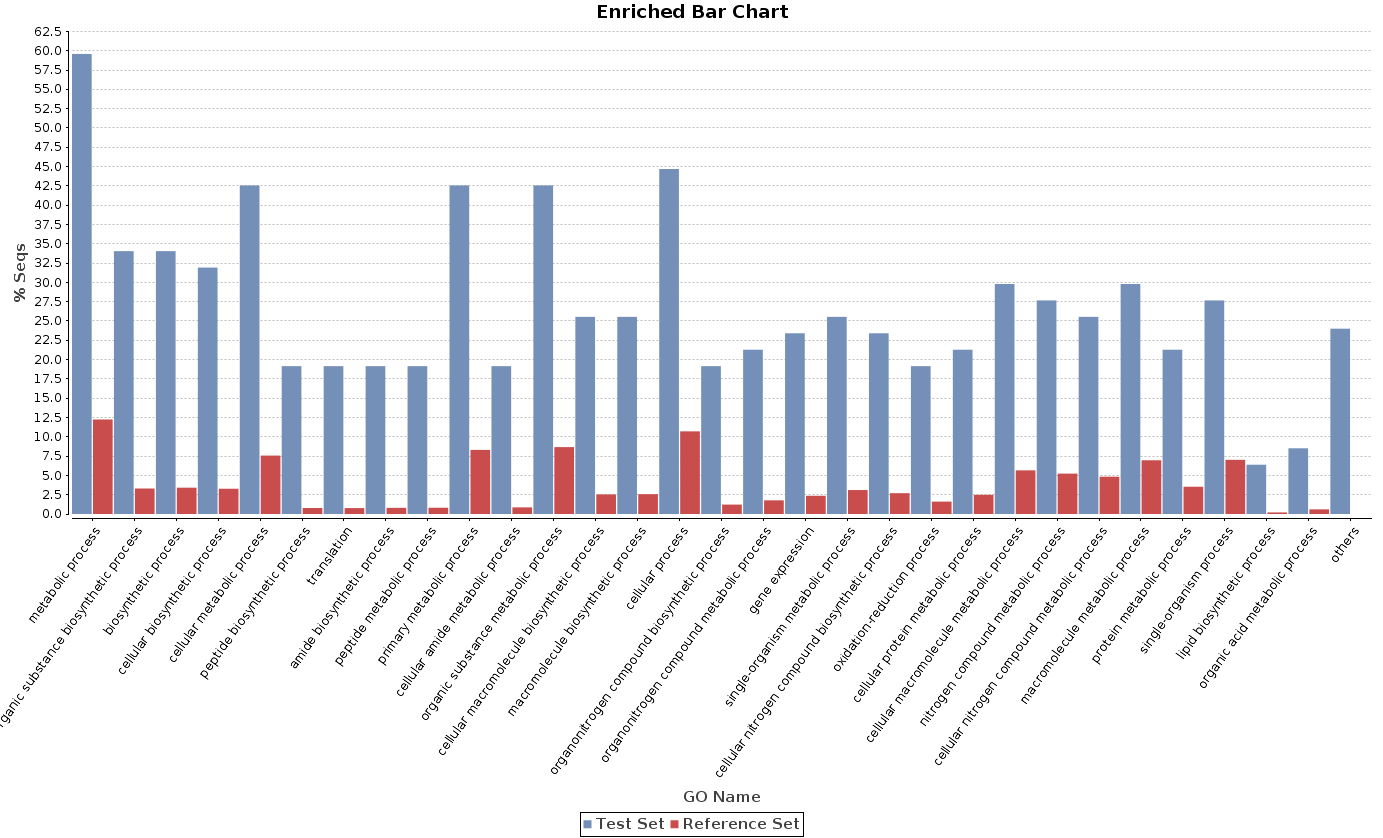


**Supplementary Figure S12.** Functional categories of genes up-regulated *only* by slavemaker *T. americanus*, while eggaged in raiding behavior (Test Set), compared to the functions of the *T. americanus* transcriptome as a whole (Reference Set).


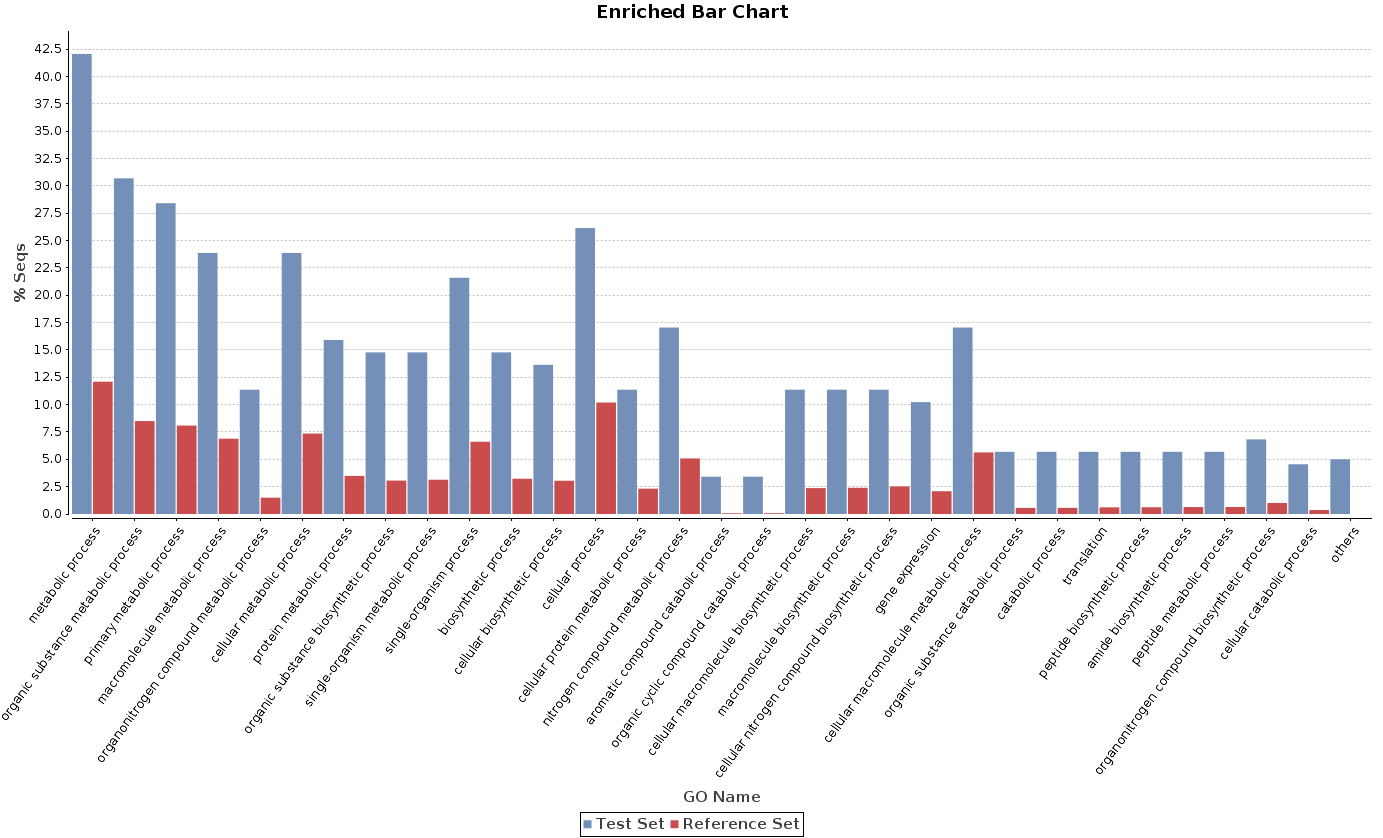


**Supplementary Figure S13.** Functional categories of genes up-regulated *only* by slavemaker *T. pilagens*, while engaged in raiding behavior (Test Set), compared to the functions of the *T. pilagens* transcriptome as a whole (Reference Set).


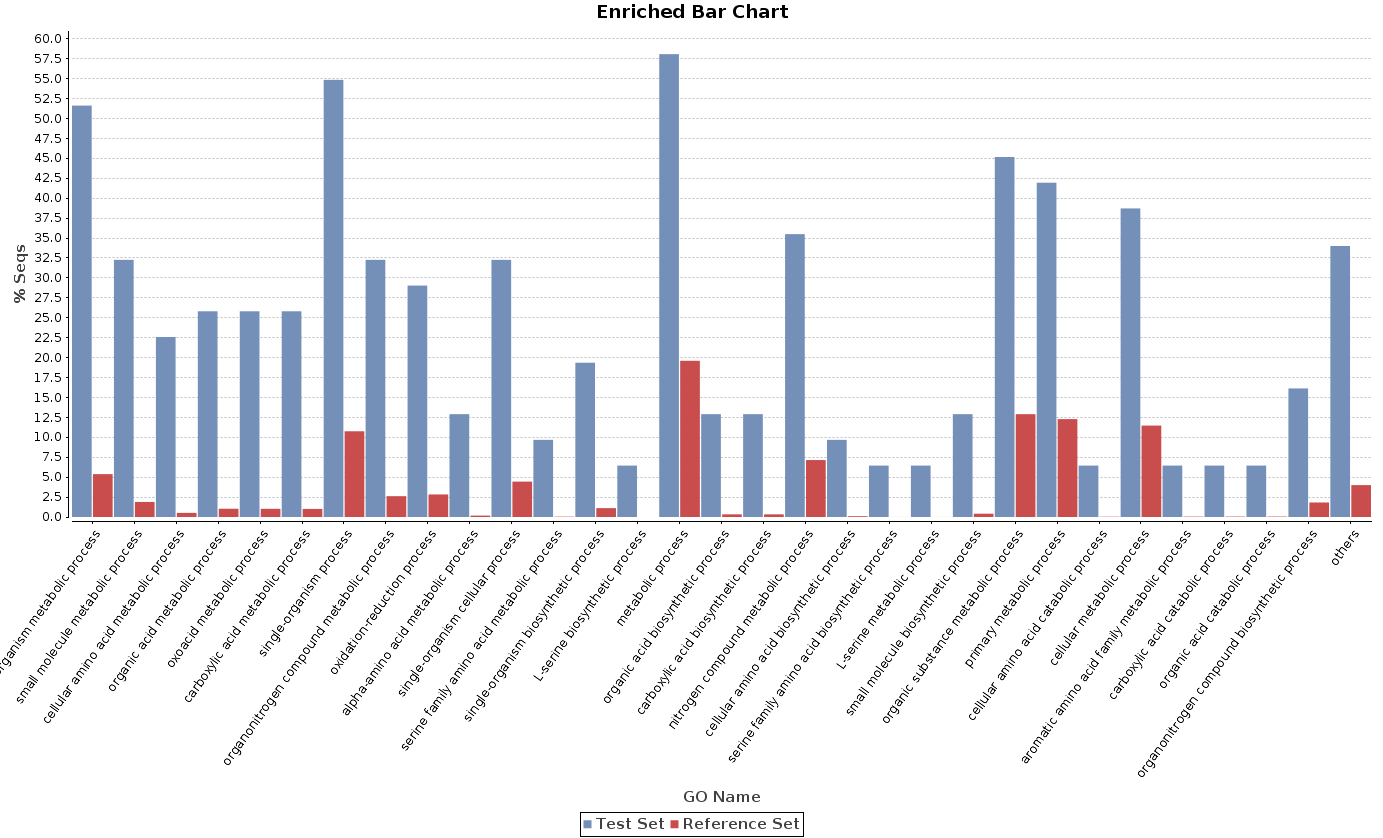


**Supplementary Figure S14.** Functional categories of *all* genes up-regulated by host *T. ambiguus*, before engaging in nest defense behavior (Test Set), compared to the functions of the *T. longispinosus* transcriptome as a whole (Reference Set).


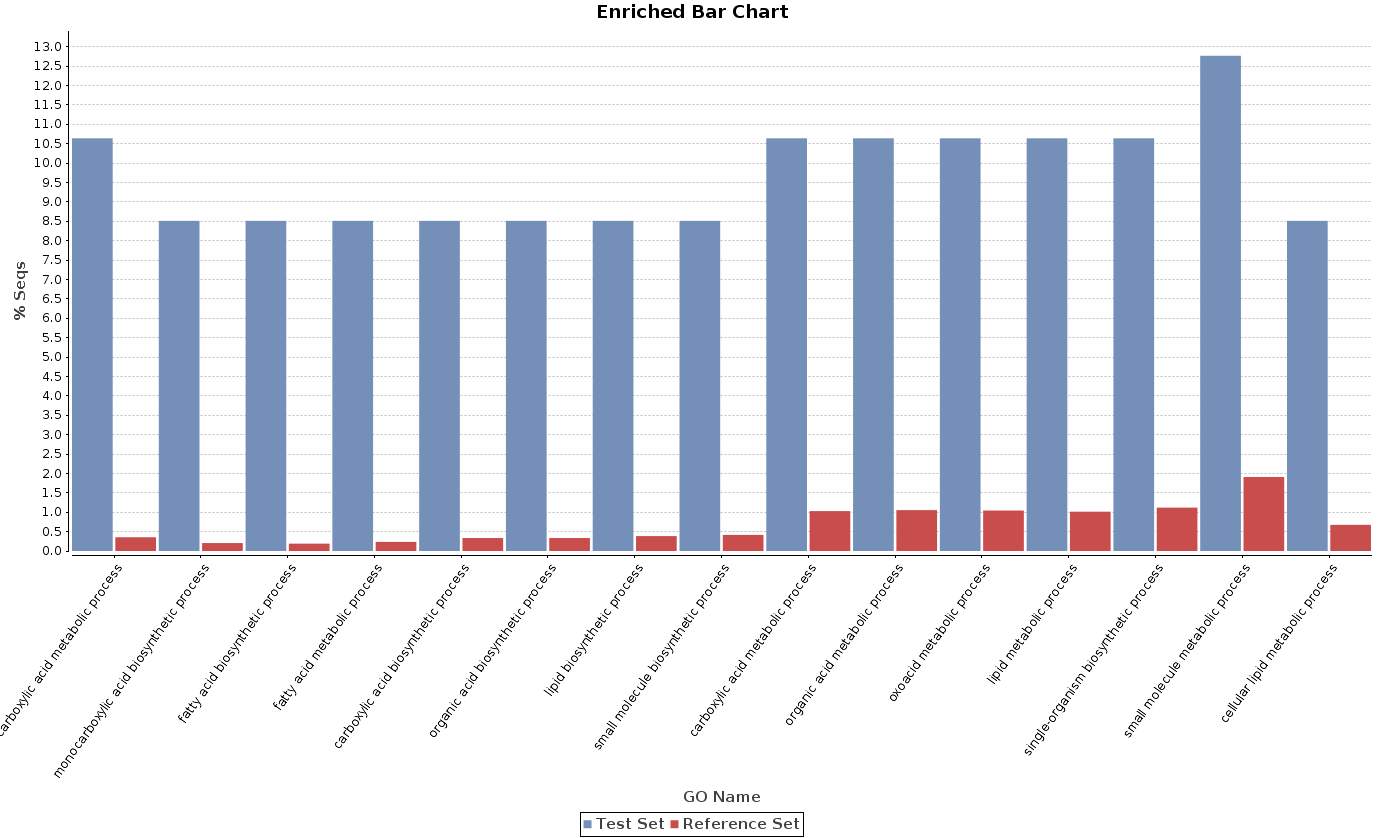


**Supplementary Figure S15.** Functional categories of *all* genes up-regulated by host *T. ambiguus*, during nest defense behavior (Test Set), compared to the functions of the *T. ambiguus* transcriptome as a whole (Reference Set).


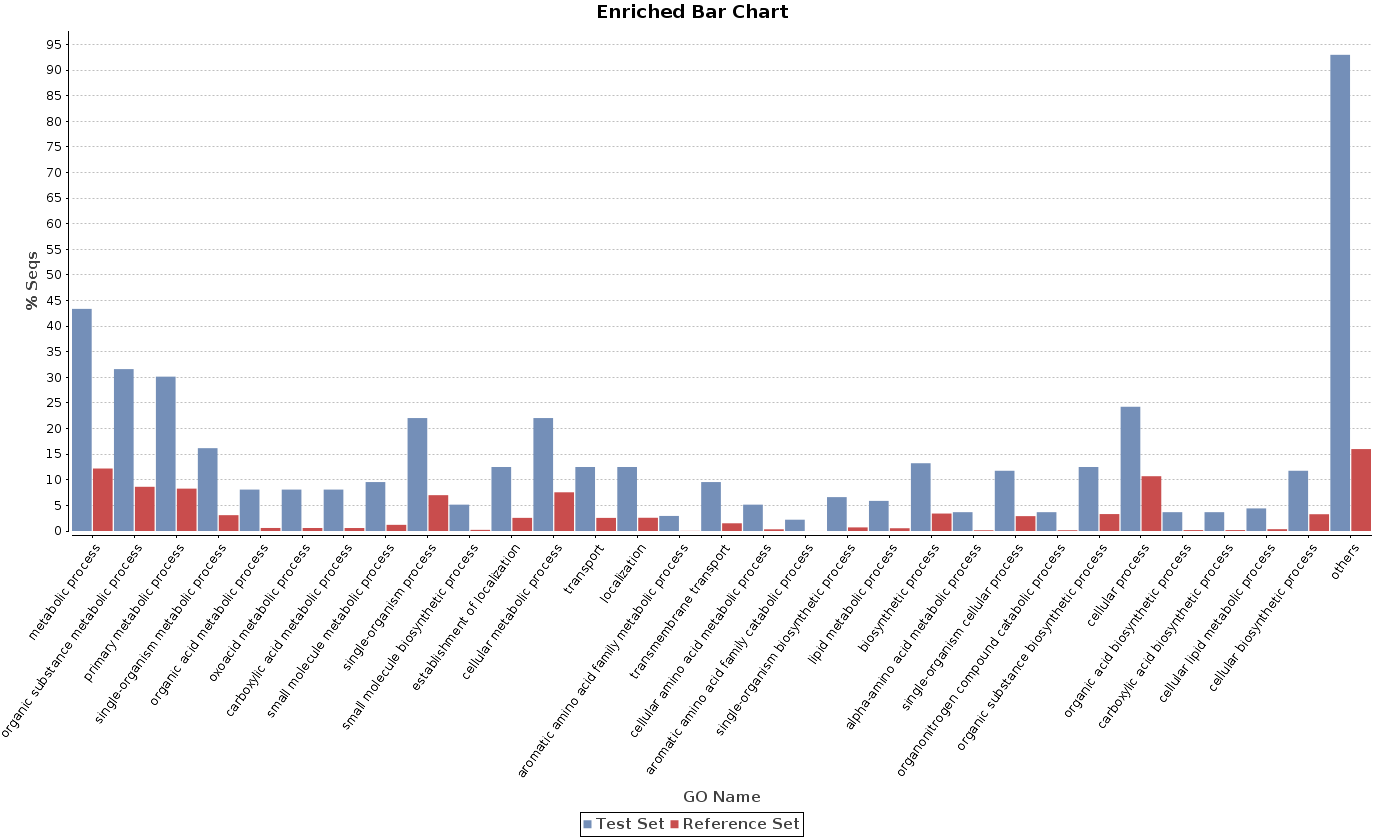


**Supplementary Figure S16.** Functional categories of *all* genes up-regulated by slavemaker *T. americanus*, while out of raiding season (Test Set), compared to the functions of the *T. americanus* transcriptome as a whole (Reference Set).


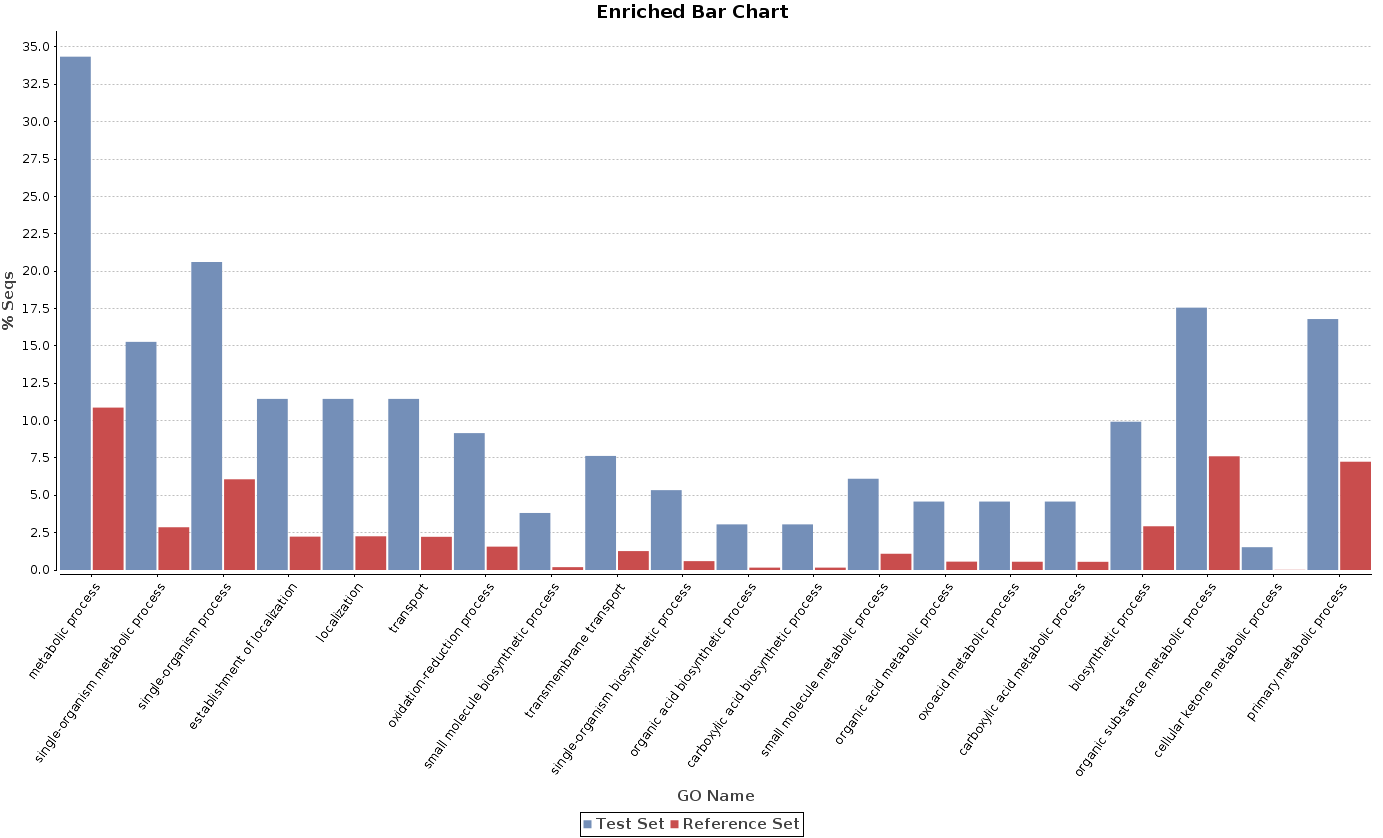


**Supplementary Figure S17.** Functional categories of *all* genes up-regulated by slavemaker *T. duloticus*, while out of raiding season (Test Set), compared to the functions of the *T. duloticus* transcriptome as a whole (Reference Set).


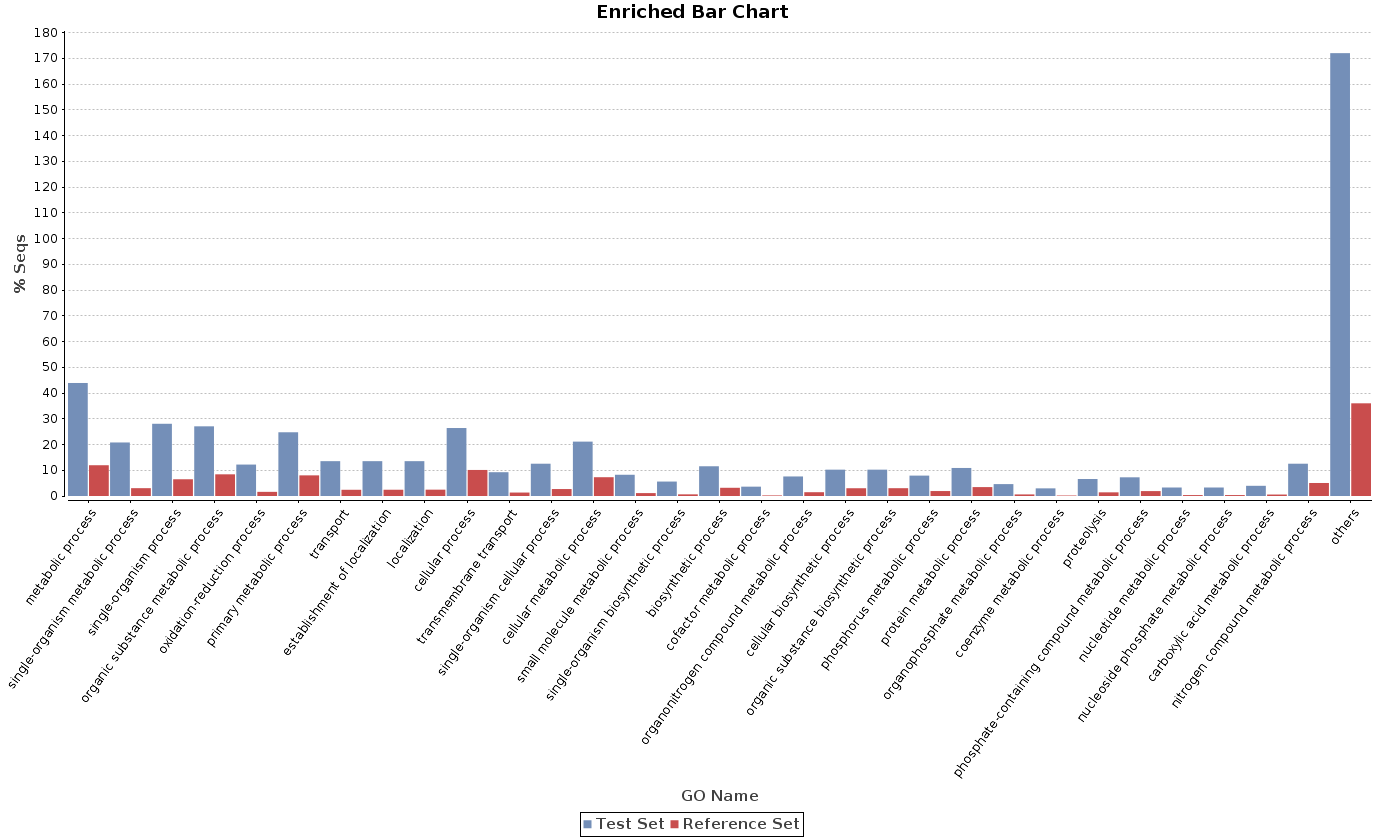


**Supplementary Figure S18.** Functional categories of *all* genes up-regulated by slavemaker *T. pilagens*, while out of raiding season (Test Set), compared to the functions of the *T. pilagens* transcriptome as a whole (Reference Set).


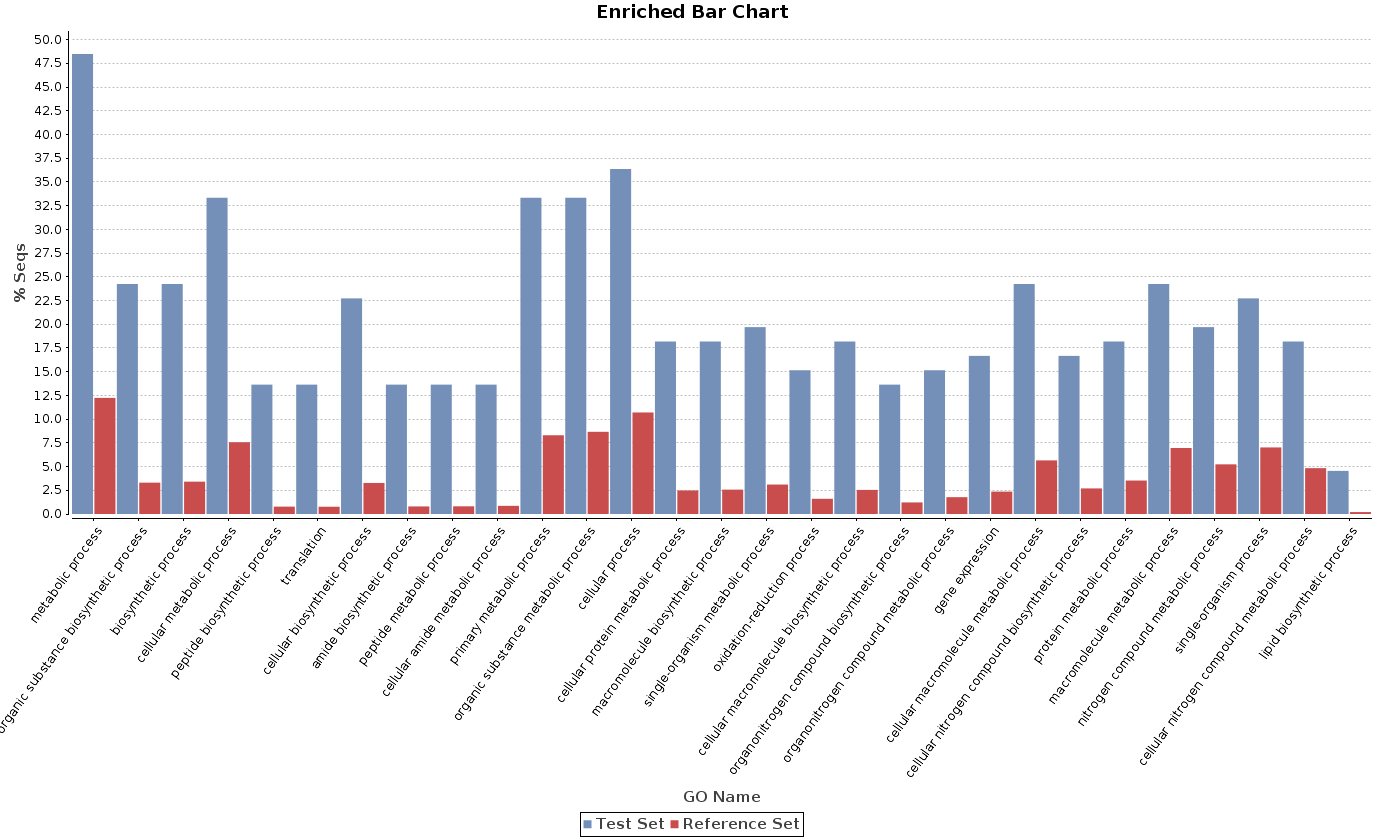


**Supplementary Figure S19.** Functional categories of *all* genes up-regulated by slavemaker *T. americanus*, during raiding behavior (Test Set), compared to the functions of the *T. americanus* transcriptome as a whole (Reference Set).


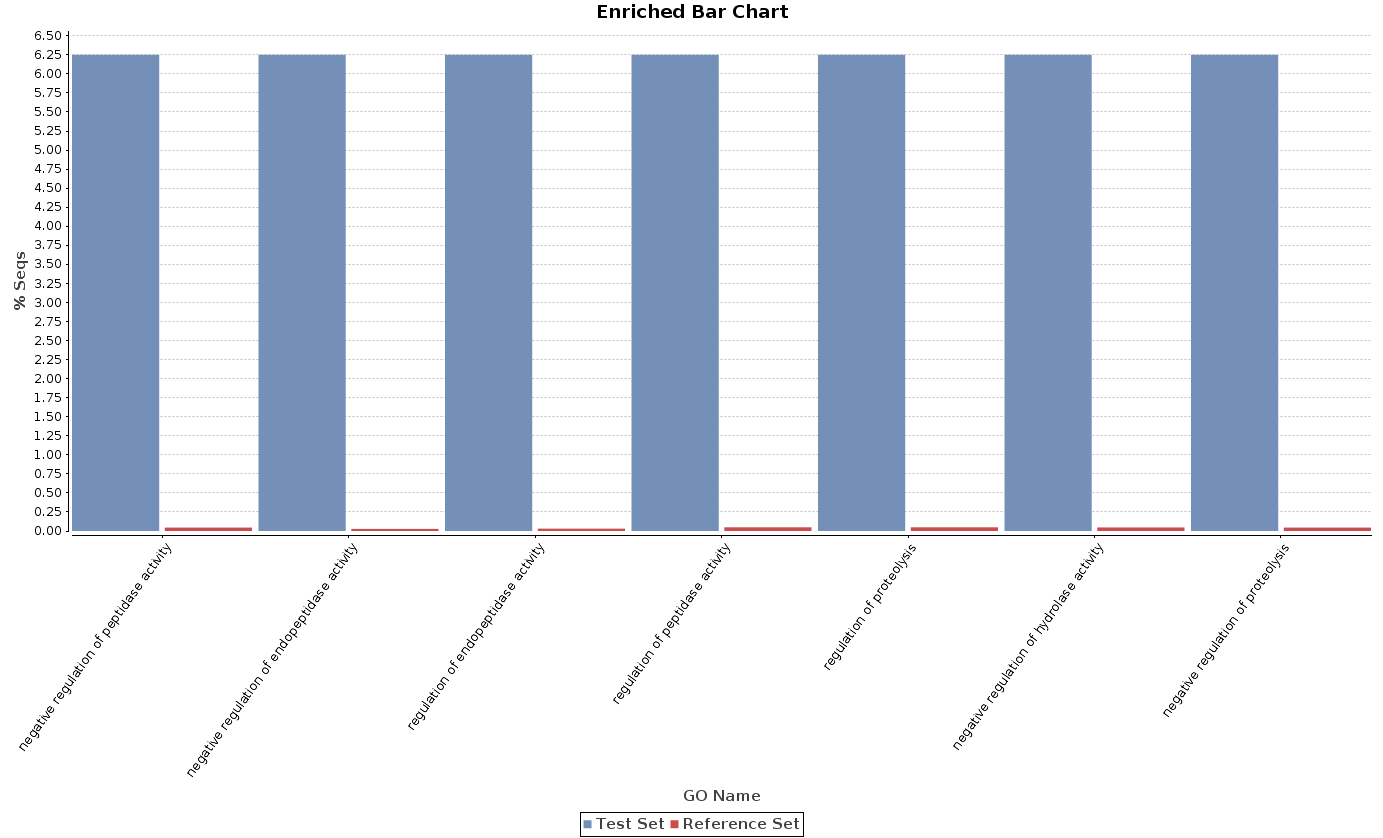


**Supplementary Figure S20.** Functional categories of *all* genes up-regulated by slavemaker *T. duloticus*, during raiding behavior (Test Set), compared to the functions of the *T. duloticus* transcriptome as a whole (Reference Set).


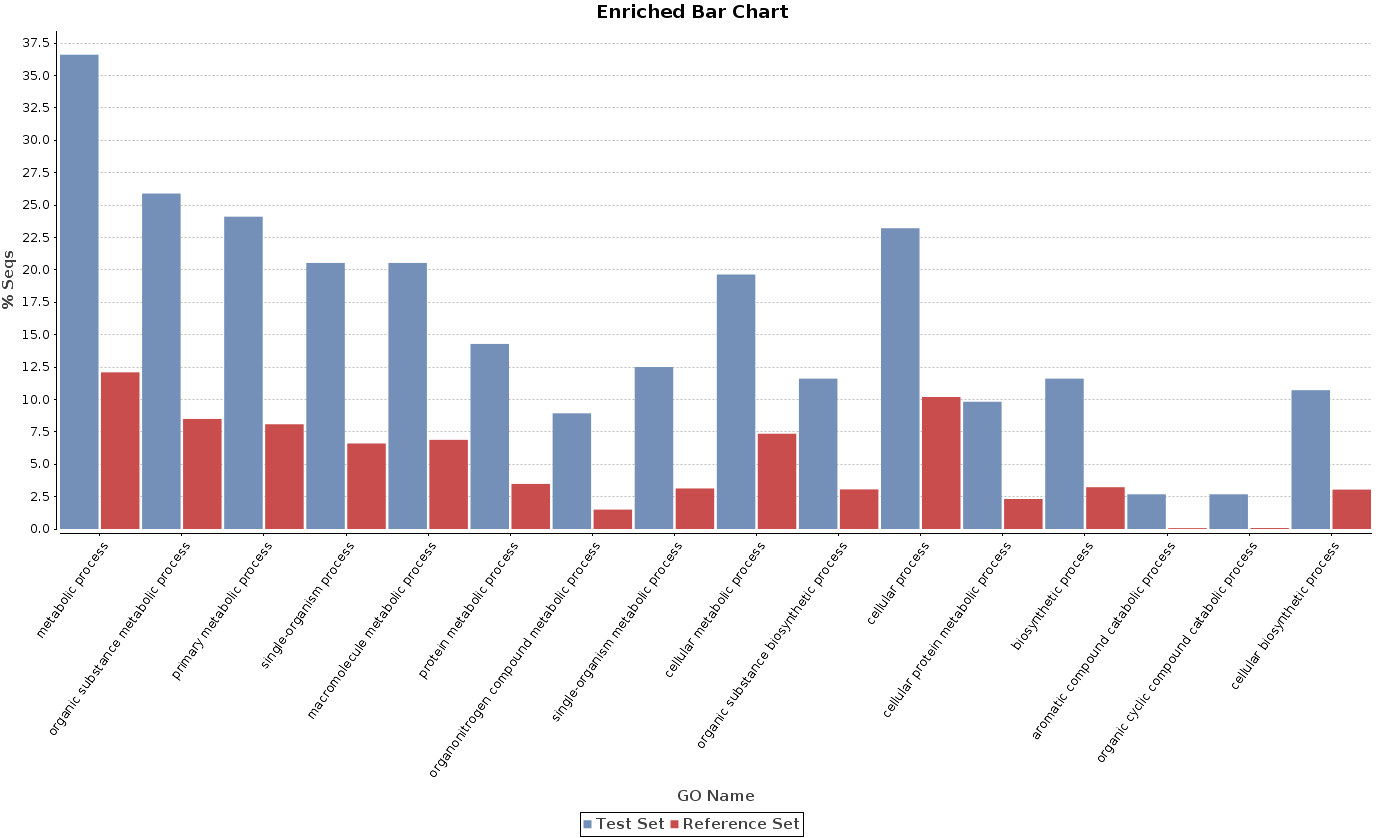


**Supplementary Figure S21.** Functional categories of *all* genes up-regulated by slavemaker *T. pilagens*, during raiding behavior (Test Set), compared to the functions of the *T. pilagens* transcriptome as a whole (Reference Set).


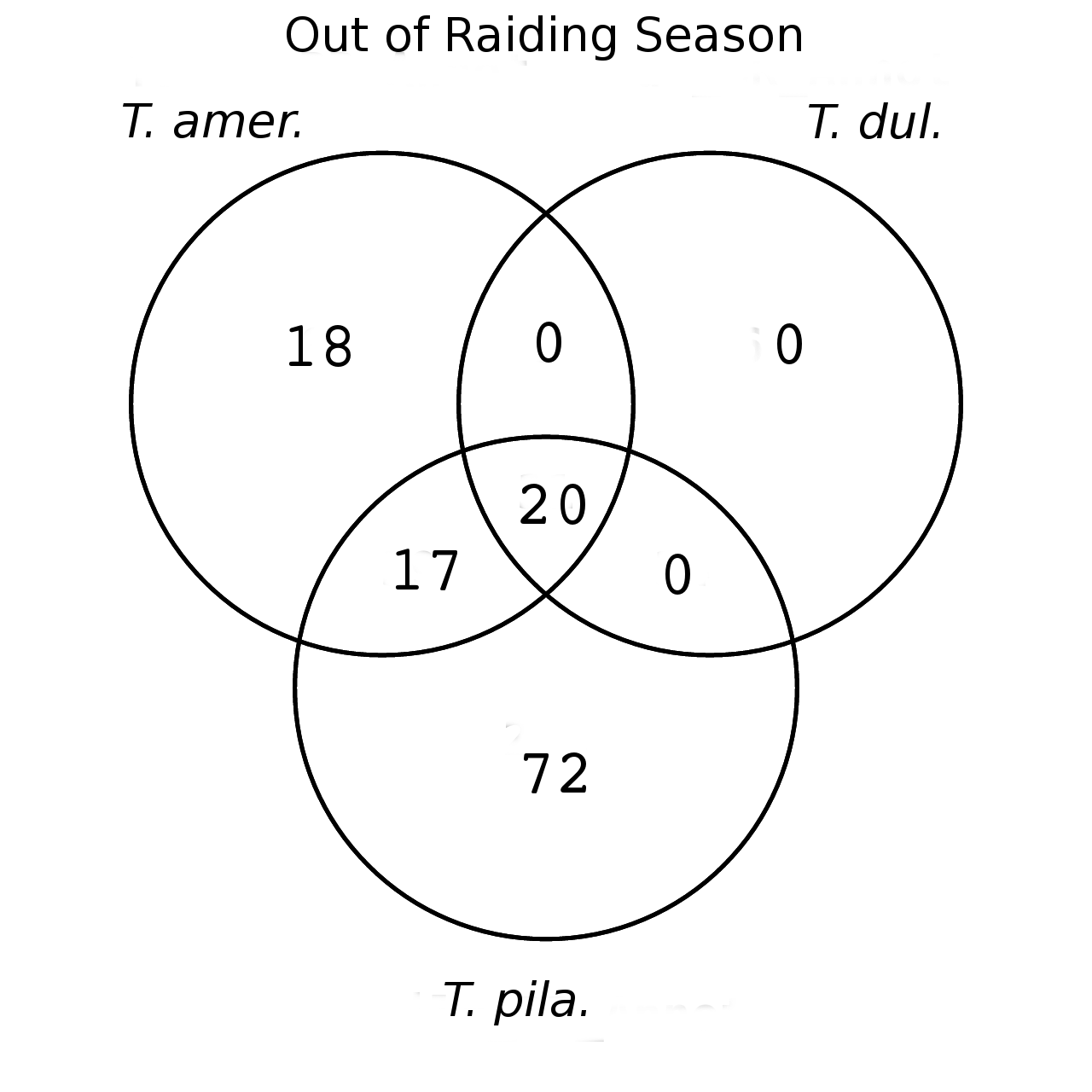


**Supplementary Figure S22.** Number of private and shared GO terms after functional enrichment of slavemaker clusters out of raiding season.


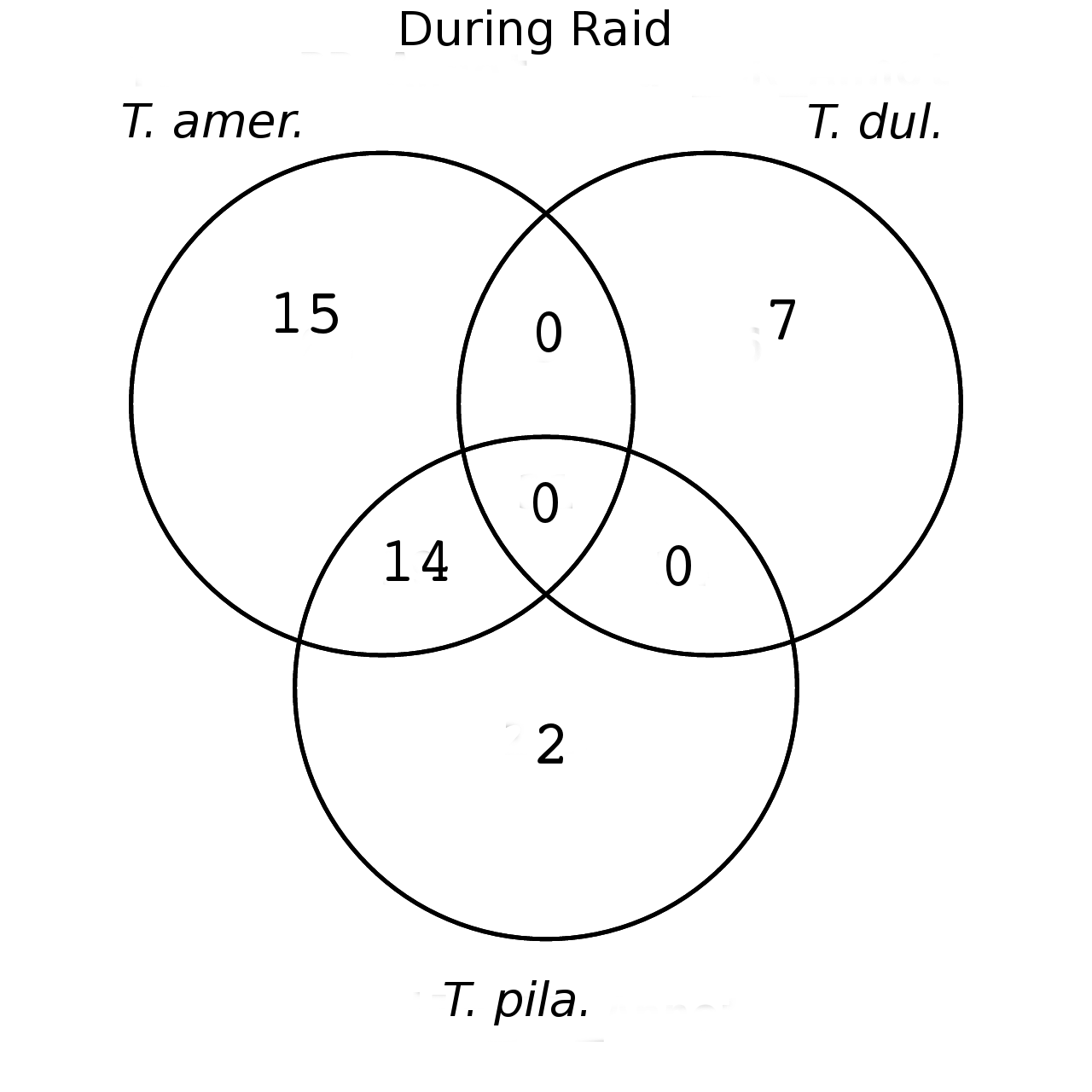


**Supplementary Figure S23.** Number of private and shared GO terms after functional enrichment of slavemaker clusters during the raiding state.

# Supplementary Table S9. Sampling year and location of collection sites for all individuals used for RNA-Seq.

| **Collection Year** | **Slavemaker Species** | **Host Species** | **State** | **Site** | **Latitude** | **Longitude** |
| --- | --- | --- | --- | --- | --- | --- |
| 2012 | *T. americanus* | *T. longispinosus* | New York | Huyck Preserve - Border | 42.533500 | -74.163533 |
|  |  |  |  | Huyck Preserve - Dam | 42.517350 | -74.056717 |
|  |  |  |  | Huyck Preserve - Oadway Trail | 42.532483 | -74.145883 |
|  |  |  |  | Huyck Preserve - Inter-swamp | 42.530783 | -74.145350 |
| 2012 | *T. duloticus* | *T. curvispinosus* | Ohio | Highbanks | 40.090568 | -83.022036 |
|  |  |  |  | Orentangy Indian Fauk | 40.191167 | -83.064283 |
|  |  |  |  | Alume Creek, Campground | 40.237117 | -82.985150 |
| 2013 | *T. pilagens* | *T. ambiguus* | Michigan | Michigan_A | 44.768850 | -86.074933 |
|  |  |  |  | Michigan_B | 44.764833 | -86.074317 |
|  |  |  |  | Michigan_C | 44.765900 | -86.074367 |
|  |  |  |  | Michigan_D | 44.755967 | -86.071117 |
